# Development and Properties of Valine-Alanine based Antibody-Drug Conjugates with Monomethyl Auristatin E as the Potent Payload

**DOI:** 10.3390/ijms18091860

**Published:** 2017-08-25

**Authors:** Yanming Wang, Shiyong Fan, Wu Zhong, Xinbo Zhou, Song Li

**Affiliations:** 1 School of Pharmaceutical Engineering, Shenyang Pharmaceutical University, Shenyang 110016, China; yanming0117@163.com; 2 Laboratory of Computer-Aided Drug Design & Discovery, Beijing Institute of Pharmacology and Toxicology, Beijing 100850, China; fsyn1996@163.com (S.F.); zhongwu@bmi.ac.cn (W.Z.)

**Keywords:** antibody-drug conjugate, epithelial growth factor receptor, valine-alanine, monomethyl auristatin E

## Abstract

Antibody-drug conjugates (ADCs), designed to selectively deliver cytotoxic agents to antigen-bearing cells, are poised to become an important class of cancer therapeutics. Human epithelial growth factor receptor (HER2) is considered an effective target for cancer treatment, and a HER2-targeting ADC has shown promising results. Most ADCs undergoing clinical evaluation contain linkers that have a lysosomal protease-cleavable dipeptide, of which the most common is valine-citrulline (VC). However, valine-alanine (VA), another dipeptide comprising two human essential amino acids, has been used in next generation ADCs loading new toxins, but the druggable properties of ADCs loaded the most popular monomethyl auristatin E (MMAE) remain to be further explored. In this study, we generated VA-based ADCs that connected MMAE to an anti-HER2 antibody. We studied the differences in the preparation process, in vitro stability, cathepsin B activity and in vitro cytotoxicity of VA-based ADC compared to the ADC of VC. VA had comparable performance to VC, which preliminarily displays its practicability. Additional efficacy and safety studies in a xenograft model indicate this novel ADC exerted potent anti-tumor activity and negligible toxicity. The results of this study show the application potential of VA-based ADC with MMAE as the payload.

## 1. Introduction

Human epithelial growth factor receptor 2 (HER2/ErbB2) is a ligand-less tyrosine kinase receptor that acts as a pro-oncogene in different human cancers [1]. HER2 plays an important role in the development and prognosis of many cancer types such as metastatic breast, gastric, lung, colon, esophageal, and ovarian cancers [2,3]. For instance, it is overexpressed in approximately 20–25% of breast cancers, which were characterized by aggressive proliferation and poor prognosis before the introduction of targeted treatments [4,5]. HER2 is considered an effective target for antibody-based therapy in cancer treatment, especially breast cancer [6,7]. Trastuzumab (Herceptin^®^) and pertuzumab (Perjeta^®^) are two approved monoclonal antibody drugs that target the HER2 pathway, and their clinical application has greatly prolonged the survival of patients with metastatic breast cancer [8,9]. Despite these noteworthy advances in HER2-targeted therapy, patients eventually relapse, underscoring the need for alternative therapies [10].

Antibody-drug conjugates (ADCs), which combine the specificity, pharmacokinetics, and biodistribution of a monoclonal antibody (MAB) with the cytotoxic potency of a drug payload, is a promising new therapy for cancer [11]. Ado-trastuzumab emtansine (Kadcyla^®^), an anti-HER2 ADC that was designed with this new strategy, further prolonged the overall survival of trastuzumab- resistant patients by more than six months [10,12]. The three components of ADCs are in the form of a targeted drug delivery system [13]. In addition to the development of antibodies and cytotoxic drugs, the design of the linker is of essential importance, as it impacts the efficacy and tolerability of the final product.

**Figure 1:**
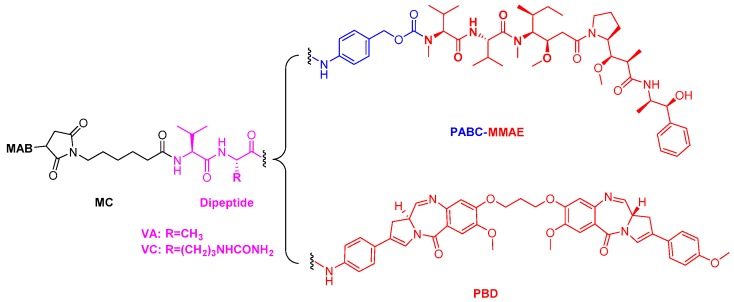
Structure models of the antibody-drug conjugates (ADCs): MAB-MC-VC-PABC-MMAE and MAB-MC-VA-PBD. MAB: monoclonal antibody; MC: maleimidocaproyl; VC: valine-citrulline; VA: valine-alanine; PABC: p-aminobenzylcarbamate; PBD: pyrrolobenzodiazepine; MMAE: monomethyl auristatin E.

A suitable linker not only needs to provide sufficient stability during systemic circulation, but must also allow the rapid and efficient release of cytotoxic drug inside the tumor cells [14]. First-generation ADCs often contained unstable linkers, such as unhindered disulfides and hydrazones, which led to their failure [11,15]. On the other hand, next-generation ADCs contain linkers such as peptide linkers, glucuronides, and noncleavable linkers, which increase their stability [16,17]. Cleavage of the commonly used dipeptide linker depends on tumor-associated lysosomal cathepsin B and undergoes rapid hydrolysis, leading to release of the parent drug to kill the tumor [18,19]. The majority of auristatin-based ADCs use a dipeptide (valine-citrulline (VC)) linker conjugated to the auristatin analog monomethyl auristatin E (MMAE) [20,21]. In recent years, valine-alanine (VA), another dipeptide comprising two human essential amino acids, has been used in next generation ADCs loading new toxins such as pyrrolobenzodiazepine (PBD) [22,23]. In addition, the VA-based linker also been mentioned in patents for a novel internalizing ADC that connected MMAE to antibody via a dibromo-containing linker, with any relevant druggable properties were provided [24,25]. The effect of different cytotoxin on the ADC is very significant, in addition to PBD and MMAE being two completely different molecules with different drug release process (Figure 1) [14,26]. The VA peptide has an advantage in the preparation process and cost of materials, and may have the potential to improve ADC stability and decrease product aggregation [27,28]. This has led to interest in exploring the physical and chemical properties, stability, enzymolysis kinetics, and efficacy of VA-based ADCs with MMAE as the potent payload.

In this study, we generated VA-based ADCs that connected MMAE to an anti-HER2 antibody. Compared to the VC-based ADC which payload been from a clinically approved ADC drug, we studied the preliminarily practicability of the VA-based ADCs in the preparation process, in vitro stability, cathepsin B activity and in vitro cytotoxicity. In addition, we further studied the anti-tumor activity and toxicity of VA-based ADC in a xenograft model. The results demonstrate that the VA-based ADC with MMAE as the payload also has application potential as an antitumor agent.

## 2. Results and Discussion

### 2.1. Linker Design

The design of the VA-based linker system was based on acetazolamide-drug conjugates [27]. Compared to counterparts with valine-lysine or valine-arginine linkers, small molecule-drug conjugates (SMDC) with VC and VA linkers exhibited greater stability and superior therapeutic activity. The SMDC containing the VA dipeptide linker not only showed the highest stability in vitro but also the highest efficacy in vivo and in vitro. Applying the linker of this small molecule to ADCs may improve the performance of the final product. In addition, compared to the VC dipeptide, VA may have the potential to reduce ADC aggregation [28].

Both linkers with VA and VC were substrates for cathepsin B, and VA-based ADC could be easily cleaved by enzymes in an antibody-doxorubicin conjugate [28]. The VA dipeptide has also been used in clinical trials of ADCs containing PBD as the cytotoxic payload such as SGN-CD33A [22]. However, the effect of cytotoxin on the ADC is very significant, and the practicability of developing VA for MMAE-based ADC need to be further determined.

**Figure 2:**
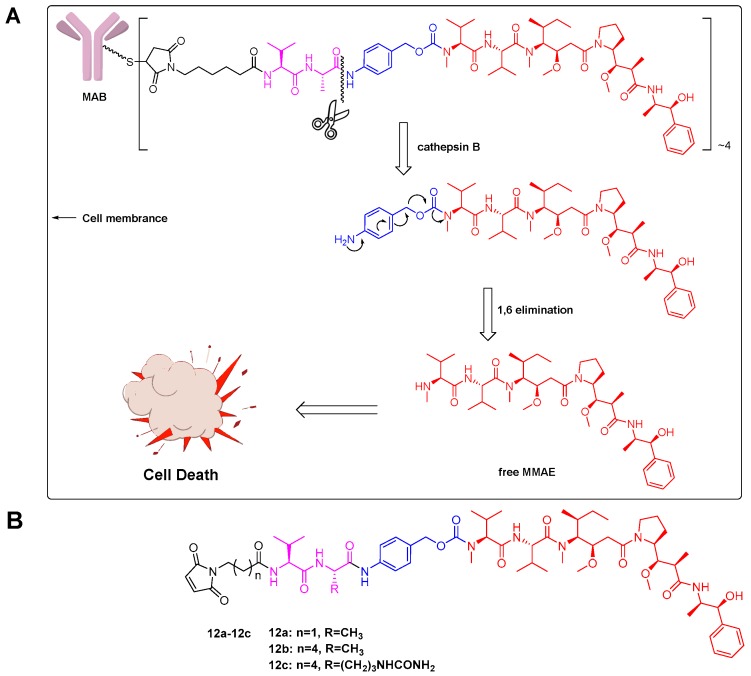
Linker design: (**A**) VA-based ADC with MMAE as the payload and its lysosomal processing. Upon internalization of ADC into the lysosomes of target cells, the amide bond between the alanine residue and the p-aminobenzylcarbamate (PABC) portion of the linker was cleaved by cathepsin B. Self-immolation of the PABC portion of the payload-linker via a 1,6-elimination process released free MMAE into the tumor cell, resulting in cell death; (**B**) Structure of the designed drug payload of MMAE-based ADCs: **12a**–**12c**.

We generated VA-based internalizing ADCs with MMAE as the payload, as well as the corresponding VC-based ADC as a comparison. Theoretically, this type of ADC can also release the parent drug in a similar manner (Figure 2A). We replaced the VC dipeptide with VA, with the goal of proving the feasibility of this design strategy. This study also explored the effects of increasing the polarity of the VA linker by shortening the carbon chain (Figure 2B). These data can be used as evidence of the effects of carbon chains on drug release performance.

### 2.2. Evaluation of Antibody-Drug Conjugates (ADC) Preparation

**Figure 3:**
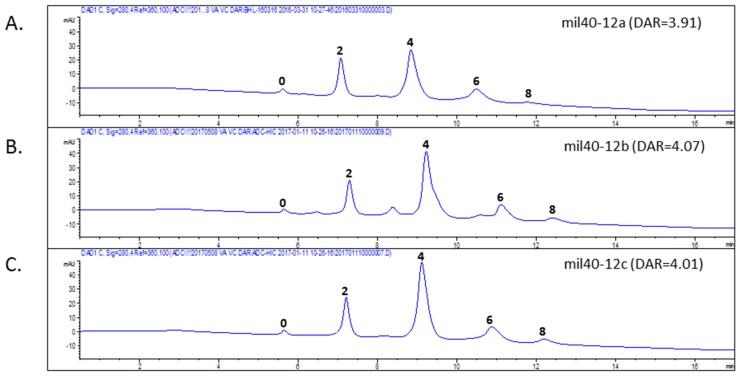
HIC analysis of ADCs. HIC allowed resolution of the conjugates into several major peaks corresponding to 0, 2, 4, 6, and 8 drug molecules per antibody. (**A**) Drug distribution of ADC mil40-**12a**, DAR = 3.91; (**B**) Drug distribution of ADC mil40-**12b**, DAR = 4.07; (**C**) Drug distribution of ADC mil40-**12c**, DAR = 4.01. DAR: drug to antibody ratio.

The drug payloads used to make ADCs are typically conjugated to the antibody through cysteine or lysine residues. The number of drug payloads per antibody, commonly referred to as the drug-to-antibody ratio (DAR), can vary between 0 and 8 drugs per antibody. Reduction of inter-molecule chain disulfide bonds to produce free sulfhydryl groups allowed conjunction at specific residues using maleimide-containing linkers, and generated conjugate mixtures at a limited number of defined positions. Hydrophobic interaction chromatography (HIC) analysis of the three ADCs (mil40-**12a**, mil40-**12b**, and mil40-**12c**; mil40 is a humanized monoclonal antibody that binds to HER2) allowed resolution of the conjugates into several major peaks corresponding to 0, 2, 4, 6, and 8 drug molecules per antibody (Figure 3), with an average DAR of about 4.

Similar to other biologics, it is important to monitor high molecular weight species in ADC. Size exclusion chromatography (SEC) is a long-established method to measure size variants of proteins, especially high molecular weight species. To confirm the effects of dipeptide type on the degree of aggregation, we synthesized two types of ADCs using VA and VC as linkers. Then, we analyzed aggregation of the two prepared ADCs with an average DAR of about 4. The dimer of the aggregated antibody peak of VA-based ADC (mil40-**12b**) was 0.27% in the high-performance liquid chromatography (HPLC) spectrum (Agilent 1260; Wilmington, DE, USA), whereas that of VC-based ADC (mil40-**12c**) was 0.83%. To enlarge this effect, we synthesized the two ADCs with an average DAR of about 7. In these samples, VA-based ADC had no obvious increase in dimeric peak; however, the aggregation of VC-based ADC elevated to 1.80% with an increase in DAR from 4 to 7. In addition, the reaction system of VA-based conjugates had higher limpidity in the conjugate process, leading the recovery rate of the antibody to increase by 8.4%. Thus, VA was chosen over VC to reduce aggregation in ADCs with MMAE as the potent payload.

### 2.3. Stability Assays In Vitro

**Figure 4:**
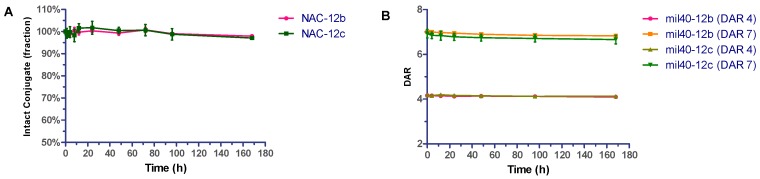
Stability assays of VA- and VC-based conjugates in PBS. Compound in phosphate buffered saline (PBS) (pH 7.4) were incubated at 37 °C for seven days. Error bars represent standard deviation from three independent experiments (triplicates). Results are shown as the mean ± SD. (**A**) Stability of NAC-**12b** and NAC-**12c**; (**B**) Stability of VA- and VC-based ADCs loaded with four and seven drugs per antibody.

ADCs and their payloads were tested for stability in phosphate-buffer saline (PBS). To compare the linker stability of the two dipeptides, the reactive maleimide double bond of **12b** and **12c** were reduced with excess *N*-acetyl-l-cysteine (NAC) to afford NAC-**12b** and NAC-**12c** respectively [29]. These compounds were added to PBS buffer and incubated at 37 °C for seven days. Aliquots were taken at various time points and stored at −20 °C before HPLC analysis. Results were based on the area under the curve (AUC) of NAC-**12b** and NAC-**12c** at each time point as a percentage of the AUC at *t* = 0. After seven days, the AUCs for NAC-**12b** and NAC-**12c** were 97.92% and 97.05%, respectively, of the two samples taken immediately at *t* = 0 (Figure 4A). In addition, it could be seen from the HPLC spectrum that the lost fraction was converted to the ring-opened succinimide hydrolysis adduct.

Next, we analyzed the in vitro stability of these two ADCs with an average DAR of about 4 and 7. Analogously, the two ADCs having 4 and 7 drugs per antibody, respectively, were diluted with PBS (1 mg/mL, pH 7.4) and incubated at 37 °C in a shaking incubator. All of the samples were analyzed by HIC HPLC. As shown in Figure 4B, none of the four ADCs had significant drug off-targets in this test, and VA- and VC-based ADCs that having four payloads decreased stability by 1.58% and 1.34%, respectively, after seven days, whereas those that having seven payloads decreased stability by 2.52% and 3.88%, respectively, under the same test conditions. In short, ADCs with VA- and VC-based linker systems have substantially comparable in vitro stability in PBS.

### 2.4. Cathepsin B Reactivity

**Figure 5:**
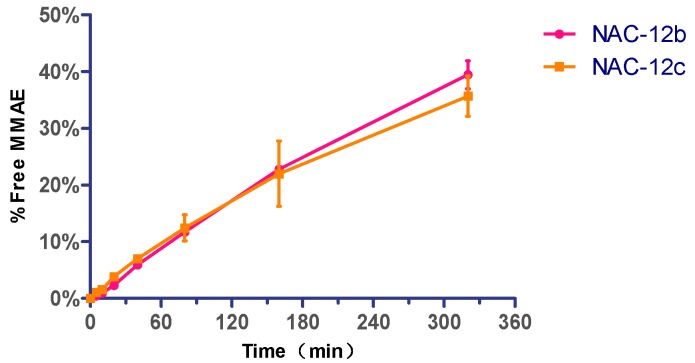
Cathepsin B reactivity for MMAE release. Typical time course of MMAE cleaved from *N*-acetyl-l-cysteine (NAC) conjugates based on VA and VC dipeptides. The enzyme-to-substrate ratio was 1:1000. Error bars represent standard deviation from three independent experiments (triplicates). Data are shown as mean ± SD.

According to previous reports, drug linkers containing VA or VC dipeptides are substrates for cathepsin B [28]. Here, we confirmed the cleavable feasibility of VA-based conjugate under the reaction of cathepsin B. The susceptibility of the dipeptide linker conjugates (NAC-**12b** and NAC-**12c**) to enzymatic cleavage was determined by treatment of the acetyl-L-cysteine adduct with cathepsin B from human liver (EC 3.4.22.1, SIGMA-ALDRICH^®^, St. Louis, MO, USA); NAC-**12c** was tested in parallel for comparison. The cleavage of dipeptide from both substrates resulted in 1,6-elimination of MMAE, which was identified by liquid chromatography-mass spectrometry (LC-MS; Agilent 1100, Palo Alto, CA, USA). The amount of released MMAE over time is shown in Figure 5. These results demonstrated that the performance of VA-based conjugate is basically the same as VC, and is likely to be a suitable substrate for cathepsin B.

### 2.5. In Vitro Evaluation of Cytotoxic Agents and ADCs

**Figure 6:**
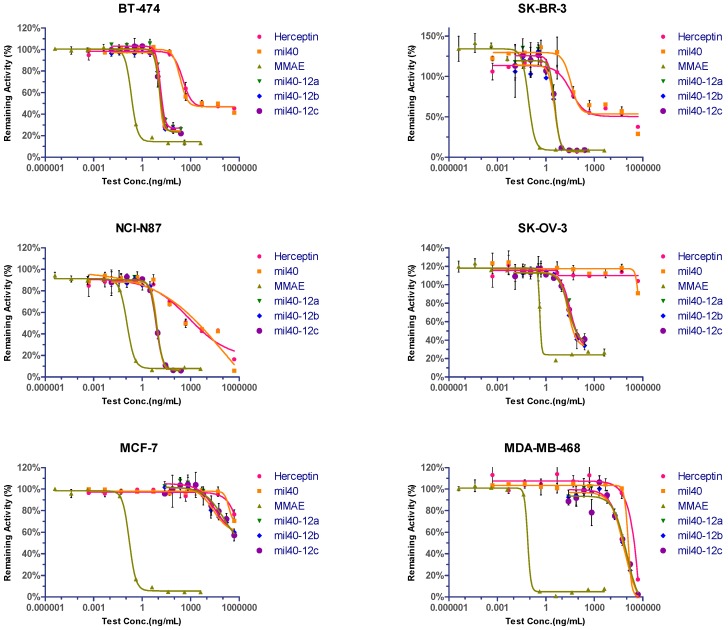
Cytotoxicity and selectivity of ADCs in tumor cells in vitro. Cytotoxicity assay performed in HER2-positive, weakly positive and negative cell lines. Breast cancer cell lines BT-474 and SK-BR-3, gastric cancer cell line NCI-N87, and ovarian cancer cell line SK-OV-3 were HER2-positive. Breast cancer cell lines MCF-7 and MDA-MB-468 were HER2-weakly positive and -negative, respectively. Each group was established three holes and results are shown as mean ± SD. IC_50_ is presented on Table 1.

To compare in vitro potency, the HER2-positive cell lines (BT-474, SK-BR-3, SK-OV-3, and NCI-N87), the weakly positive cell line MCF-7 (also known as a cell line expressing normal level of HER2) [30], and the HER2-negative cell line MDA-MB-468 were treated with various ADCs with an average DAR of 4. Both the naked antibodies (mil40 and trastuzumab) had relatively lower inhibitory effects on all HER2-positive cells, and both of them have almost the same activity in all of the tested cell lines. Meanwhile, the three ADCs (mil40-**12a**, mil40-**12b**, and mil40-**12c**) exhibited more potent activity in the HER2-positive cell line, both IC_50_ and inhibition, while very weak or no inhibitory effects in the HER2-negative cell line, just as antibody drugs, indicating its significant antigen selectivity. As a comparison, MMAE had potent anti-tumor activity in both HER2-positive and HER2-negative cell lines (Figure 6).

The above-mentioned three ADCs had almost identical cytotoxicity in vitro, indicating that the type of dipeptide and length of the carbon chain did not have significant effects on cytotoxicity and drug release in cells. Overall, VA-based ADCs with MMAE exhibited similar efficacy and selectivity to the VC-based ADC, and the selectivity between HER2-positive and HER2-negative cells increased by about 2400–26,000 fold. In addition, both antibodies exhibited very weak cell inhibitory activity in the HER2-positive ovarian cancer cell line SK-OV-3; however, the associated ADCs exhibited similar cytotoxicity to the other cell lines, with an increase in anti-tumor activity of tens of thousands (Table 1).

### 2.6. In Vivo Evaluation

**Figure 7:**
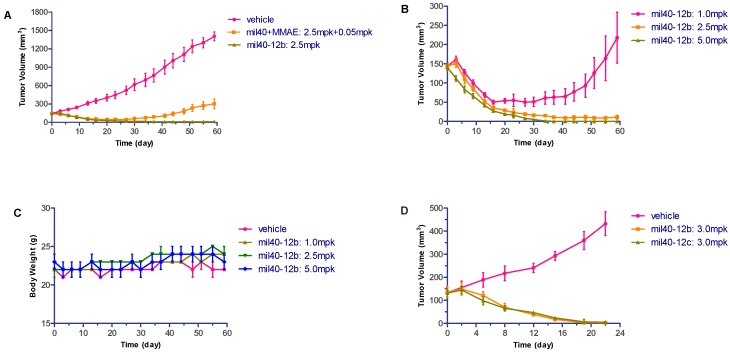
Xenograft studies of VA-based ADC (mil40-**12b**). NOD/SCID mice were implanted subcutaneously with BT-474 cells. When the tumors reached ~150 mm^3^, the animals were given vehicle, the mixture of mil40 and MMAE, and ADCs on: Days 0, 7, 14, and 21 (**A**–**C**); or Days 0, 7, and 14 (**D**). Results are shown as mean ± SD, *n* = 6/group. (**A**) Comparison of the efficacy of ADC in the middle dose group with the control group; (**B**) dose-efficacy dependence of the ADC treatment groups; (**C**) changes in body weight of the mice during the observation period; (**D**) comparison of the efficacy of ADCs based on VA and VC dipeptides.

Carrying more payloads usually speeds up the elimination of ADC in vivo [2,31]. Here, the anti-HER2 antibody of mil40 conjugated with an average of four VA-MMAE drug payloads was selected for further in vivo characterization and evaluation. The ADC (mil40-**12b**) was evaluated in the BT-474 (HTB-20™; ATCC, Maryland, USA) breast tumor model in female non-obese diabetes / severe combined immunodeficiency (NOD/SCID) mice, with naked antibody and unconjugated MMAE as the control group. Compared with vehicle, the control group only caused a partial delay in tumor growth, whereas the ADC treatment group with the same therapeutic dose exhibited very obvious tumor inhibition (Figure 7A). In the ADC medium dose treatment group (2.5 mg/kg), tumors in half of the tumor-bearing animals disappeared (3/6 cures), while all the tumors can still be observed after treatment in the vehicle or mixture treatment group. Furthermore, ADC treatment groups showed significant dose-efficacy dependence, and tumor in all tested animals at 5 mg/kg dose were all disappeared after treatment and no recurrence occurred after one month (Figure 7B). In addition, there was no weight loss observed in all groups treated with ADCs, indicating that the treatments were preliminarily well tolerated (Figure 7C). These results indicate that this VA-based ADC has potent anti-tumor activity and basic safety. Furthermore, the tumor suppression data of ADCs with VA and VC dipeptides showed that VA-based ADC had substantially comparable activity to the ADC of VC (Figure 7D), also without obviously weight loss in all treatment groups (data not shown).

### 2.7. Hematological Analysis

**Figure 8:**
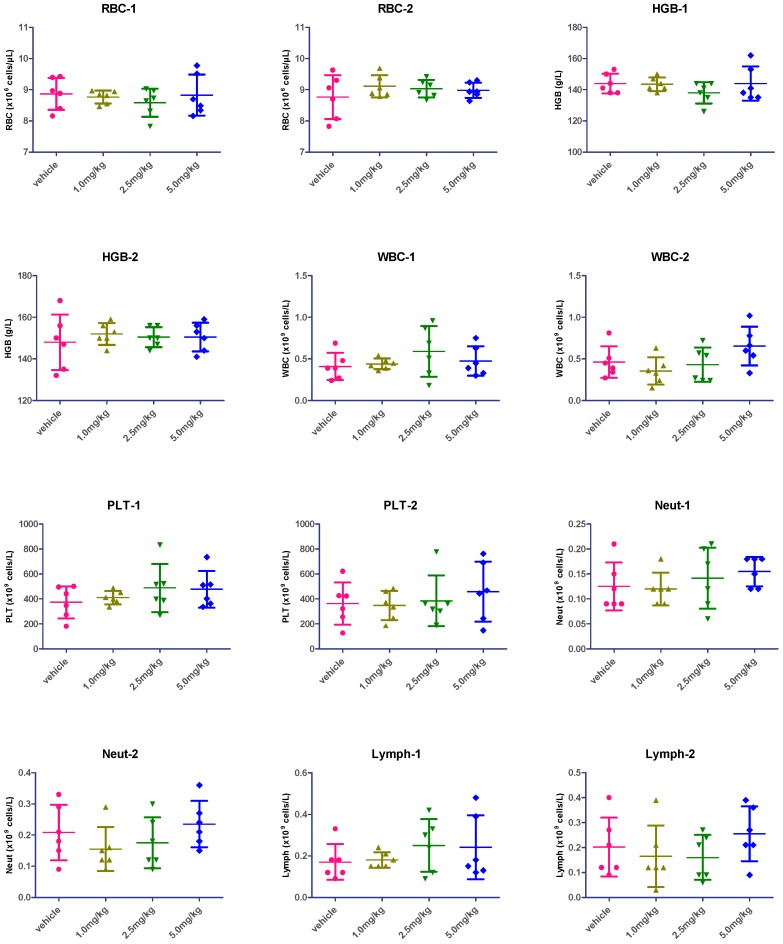
Hematological analysis of VA-based ADC (mil40-**12b**). Numbers “**1**” and “**2**” represent the first and second sampling on Days 28 and Days 58, respectively. Red blood cells (RBCs), hemoglobin (HGB), white blood cells (WBCs), platelets (PLTs), neutrophils (Neuts) and lymphocytes (Lymph) were analyzed. Results are shown as mean ± SD, *n* = 6/group.

To further verify the safety of the ADC (mil40-**12b**), changes in hematology between vehicle and treatment groups were compared at the end of administration and the observation period. The main test indicators included levels of white blood cells (WBCs), red blood cells (RBCs), platelets (PLTs), neutrophils (Neuts), and lymphocytes (Lymph). In contrast to the vehicle group, all of the above hematological parameters from the three ADC treatment groups were similar on Days 28 and 58 (Figure 8).

Analysis of variance (ANOVA) analysis was conducted on the hematology parameters of the treatment groups on Days 28 and 58, and at the end of drug administration. Unpaired two-tailed *t*-test for multiple comparisons was used. The level of confidence intervals was set at 95%, and the significance was set at *p* < 0.05. There was no significant difference between vehicle and ADC treatment groups (1, 2.5, and 5 mg/kg) regarding all parameters from both batches of blood samples (Table 2). Thus, this ADC had low blood toxicity and off-target bone marrow toxicity, which may be the reason it was well tolerated.

### 2.8. Histopathological Studies

**Figure 9:**
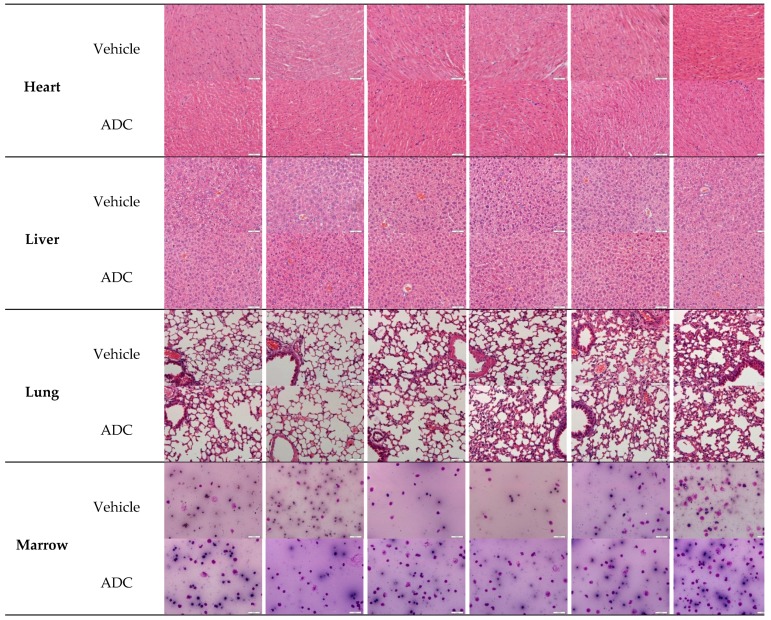
Histopathological studies of VA-based ADC (mil40-**12b**). At the end of the xenograft test, animals (*n* = 6/group) in the vehicle and ADC treatment group (2.5 mg/kg) were sacrificed, and the tissue and bone marrow were used for histopathological studies. Scale bar: 46:1; the actual size of each picture is 425 × 320 μm.

At the end of the xenograft model test, animals in specific groups were used for histopathological studies. Hematoxylin and Eosin (H&E) staining (heart, liver, and lung) and bone marrow smears showed no significant differences in histopathology evaluation, revealing no significant tissue damage produced in animals that received ADC relative to vehicle (Figure 9). This further confirms that this type of VA-based ADC has low systemic toxicity.

## 3. Materials and Methods

### 3.1. Chemistry

Unless otherwise indicated, all anhydrous solvents were commercially obtained and stored in Sure-seal bottles under nitrogen. All of the other reagents and solvents were purchased as the highest grade available and used without further purification.

Thin layer chromatography (TLC) was performed using pre-coated silica gel plates (Yantai dexin Bio-Technology Co., Ltd., Yantai, China). Column chromatography was performed using silica gel (200–300 mesh; Yantai Chemical Industry Research Institute, Yantai, China). NMR spectra were recorded on a JNM-ECA-400 400 MHz spectrometer (JEOL Ltd., Tokyo, Japan) using CDCl3 and DMSO-d6 as solvent. Chemical shifts are expressed in (ppm), with tetramethylsilane (TMS) functioning as the internal reference. MS was performed on an API 3000 triple-quadrupole mass spectrometer (AB Sciex, Concord, ON, Canada) equipped with a Turbo Ion Spray electrospray ionization source (AB Sciex, Concord, ON, Canada) that was used for mass analysis and detection. Analyst 1.4 software (AB Sciex, Concord, ON, Canada) was used for data acquisition.

### 3.2. Synthesis of the Linkers and ADC Payloads

Synthetic routes and analytical data are provided in the experimental protocols.

### 3.3. Bioconjugation and Purification

Humanized anti-HER2 IgG1 antibodies mil40 (10 mg/mL) in l-Histidine buffer (20 mM), pH 7.5, were treated with tris(2-carboxyethyl)phosphine hydrochloride (TCEP; 2.3 or 4.0 equivalents) at 25 °C for 90 min. To the reduced mAb was added the maleimide drug derivatives (1.2 equivalents /SH group) in ice-cold dimethylacetamide (DMAC) (5% *v/v*). After 40 min, the reactions were quenched with excess NAC (8 equivalents). The mixture was placed on ice for 30 min before buffer exchange by elution through Sephadex G25, and concentrated by centrifugal ultrafiltration. The conjugates was sterile filtered through a 0.2 μm filter under sterile conditions and stored at −80 °C before use for analysis and testing.

### 3.4. HPLC Analysis

HIC HPLC was used to determine levels of the molar ratio of drug substitution: 1260 HPLC (Agilent; Wilmington, DE, USA); Butyl-NPR column (2.5 μm, 4.6 × 35 mm, #14947, TOSOH Bioscience; Tokyo, Japan). Here, HIC buffer A was 50 mM potassium phosphate, pH 7.0, and 1.5 M ammonium sulfate; and HIC buffer B was 50 mM potassium phosphate, pH 7.0, 20% isopropanol. The gradient was 100% to 100% B over 15 min; flow rate was 1 mL/min; and UV detection wavelength was 280 nm. The DAR was determined by peak area integration according to the reported method [32].

SEC HPLC was used to determine levels of aggregation within each ADC: 1260 HPLC (Agilent; Wilmington, DE, USA); G3000SWXL analytical column (7.8 mm × 30 cm, #08541, TOSOH Bioscience; Tokyo, Japan). The SEC buffer contained 40 mM sodium phosphate and 150 mM sodium chloride (pH 7.0, 1 mL/min flow rate); and the UV detection wavelength was 280 nm. We performed a needle wash after each injection and include blank runs between each analyte. The aggregation was determined by peak area integration according to the reported method [33].

### 3.5. Linker Stability Assays In Vitro

The acetyl-l-cysteine (NAC) solution (0.41 mg/mL, PBS, pH 7.4) was added to **12b** and **12c** (500 mg/mL, DMSO). The reaction mixture was incubated at room temperature for 10 min, after which HPLC analysis revealed complete conversion to NAC-**12b** and NAC-**12c** as previously reported [34]. mixtures were incubated at 37 °C in a shaking incubator. Aliquots (200 μL) were collected at subsequent time points (0, 1, 2, 4, 8, 12, 24, 48, 72 h, and 7 days) and frozen at −20 °C. After sampling, all samples were melted at room temperature and analyzed by HPLC. Results were based on the AUC of remaining NAC-**12b** and NAC-**12c** at each time point as a percentage of the AUC at *t* = 0.

Analogously, ADCs that contained different dipeptide linkers were incubated in PBS (pH 7.4; 1 mg/mL) at 37 °C in a shaking incubator. Aliquots (200 μL) were collected at different time points (0, 4, 8, 12, 24, 48, 96, and 168 h) and then stored at −20 °C. All samples were thawed at room temperature before HIC analysis. The DAR of the samples at different time points were calculated as previously described [32].

### 3.6. Cathepsin B Reactivity

NAC-**12b** and NAC-**12c** (500 μg/mL) were prepared according to the above method and the enzymatic reaction temperature was set at 37 °C to mimic physiological conditions. The buffer mixture contained 25 mM sodium acetate, and 1 mM EDTA, at pH 5.5. To 880 μL of the buffer was added the NAC-conjugates solution respectively (100 μL) followed by a solution of Cathepsin B (Sigma: EC 3.4.22.1; #C8571; 20 μL of buffered aqueous solution), and the reaction mixture was incubated at 37 °C [29,35]. Aliquots (100 μL) were taken at subsequent time points (*t* = 0, 5, 10, 20, 40, 80, 160, and 320 min), and analyzed by LC-MS. Results of the released MMAE was quantified with the standard curve method.

### 3.7. In Vitro Cytotoxicity

The HER2-positive breast cancer cell line BT-474, SK-BR-3, gastric cancer cell line NCI-N87, ovarian cancer cell line SK-OV-3, weakly positive breast cancer cell line MCF-7 and HER2-negative breast cancer cell line MDA-MB-468 were obtained from ATCC (Manassas, VA, USA) and maintained in RPMI-1640 medium (Cellgro, Manassas, VA, USA) supplemented with 10% fetal bovine serum (Invitrogen, Carlsbad, CA, USA) and Glutamax (Invitrogen). Cells (3.3 × 10^4^ cells/mL) were added to each well of a 384-well plate after which 10 μL compound was also added to the assay plate. The plate was incubated for 4 days at 37 °C, 5% CO_2_, 95% humidity. Then the plates were incubated at room temperature for about 10 min, 40 μL CTG reagent was added to each well, and the plates were incubated for 30 min at room temperature. Luminescence was detected using the EnSpire Plate Reader, and Prism5 for Windows (Graphpad software, Inc., La Jolla, CA, USA) was used for data analysis, including IC_50_ calculations.

### 3.8. Xenograft Studies

Female NOD/SCID mice 6–8 weeks of age, with an average weight of approximately 22 g, were approved by Anikeeper Inc. Female NOD/SCID mice were inoculated subcutaneously with 1 × 10^7^ BT-474 breast cancer cells in 0.2 mL DMEM-Matrigel mixture (1:1 ratio) for tumor development. The treatment started when the mean tumor size reached approximately 150 mm^3^. The animals were given 1, 2.5, and 5 mg/kg ADC (mil40-**12b**), antibody + free MMAE, and vehicle alone on Days 0, 7, 14, and 21 or given 3 mg/kg ADCs (mil40-**12b** and mil40-**12c**) and vehicle on Days 0, 7, and 14. The animals were monitored twice weekly for body weight and tumor size. Tumor volume was calculated using the formula: TV = a × b^2^/2, where “a” and “b” are long and short diameters of a tumor, respectively. All procedures related to animal handling, care, and the treatment in this study were performed according to guidelines approved by the Institutional Animal Care and Use Committee of Pharmaron following the guidance of the Association for Assessment and Accreditation of Laboratory Animal Care under the licence number ON-CELL-XEM-06012016 on 1 June 2016.

### 3.9. Hematology

The animals in the xenograft model were subjected to whole-blood hematological analysis at the end of drug administration (28 days) and after 1 month of recovery (58 days). Then, 100 μL whole blood from each animal was drawn. Some samples were diluted 3 times for a final CBC count because of the volume limitation. ADC treatment-related changes in hematology parameters included RBC, HGB, WBC, PLT, Neut, and Lymph compared to the control, and results are expressed as mean ± SD. The data were analyzed base on the number with corrected dilution factor.

For comparison of hematological indicators, unpaired two-tailed *t*-test or two-way ANOVA for multiple comparisons was used. The level of significance was set at *p* < 0.05. Statistical analyses were performed using Prism5 for Windows (Graphpad software, Inc.).

### 3.10. Histopathology

At the end of the xenograft model test, animals in specific groups were used for histopathological studies. The organs mainly included the heart, liver, and lung. Tissue samples were fixed in 10% buffered formalin for 24 h and transferred into 70% ethanol. Dehydration through 70% ethanol, 85% ethanol, 90% ethanol, 95% ethanol × 2, 100% ethanol × 2 followed by 3 changes of xylene and then paraffin. Finally, the tissues were paraffin block embedded and sectioned. Then, H&E staining, mounting with permount, observation, and photographing under a microscope were performed in addition to bone marrow smears.

## 4. Experimental Section

### 4.1. Synthetic Routes

**Scheme 1:**
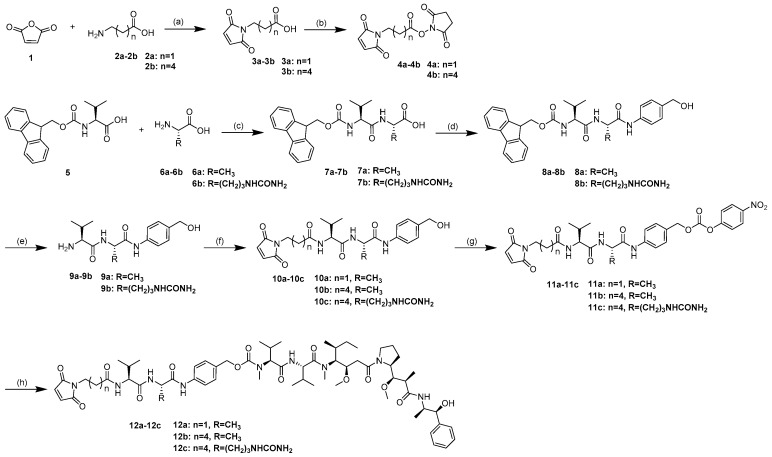
General synthetic route for synthesis of compounds **12a**–**12c**. Reagents and conditions: (**a**) AcOH, 120 °C, 6 h, 74–86%; (**b**) *N*-Hydroxysuccinimide (SuOH), TFAA, 2,4,6-trimethylpyridine, THF, 0 °C–rt, 1 h, 66–69%; (**c**) SuOH, DCC, THF, DME, rt, 2 steps, 53–71%; (**d**) p-Aminobenzyl alcohol, EEDQ, DCM/MeOH, rt, 36 h, 77–92%; (**e**) Piperidine, DMF, rt, 2 h, 84–90%. (**f**) **4**, DMF, rt, overnight, 79–87%. (**g**) Bis(4-Nitrophenyl) Carbonate, DIPEA, DMF, rt, 5 h, 86–93%; and (**h**) MMAE, HOBt, DIPEA, DMF, rt, overnight, 64–80%. rt: room temperature.

The synthetic routes for the linker-MMAE conjugates **12a**–**12c** are shown in Scheme 1.

### 4.2. Compound Synthesis and Characterization

*3-(2,5-Dioxo-2,5-dihydro-1H-pyrrol-1-yl)propanoic acid* (**3a**)*.* Maleic anhydride **1** (16.51 g, 168.37 mmol) was added to a solution of the amino acids **2a** (10.0 g, 112.25 mmol) in AcOH. The mixture was stirred at 120 °C for 6 h. The reaction mixture was poured into water after cooling to room temperature and extracted with ethyl acetate (3 × 20 mL). The organic layers were combined, washed with brine, dried over anhydrous Na_2_SO_4_, and evaporated under reduced pressure to give the crude product. Purification was performed by silica column chromatography in 1:6 ethyl acetate/petroleum ether (*v/v*). A white solid was obtained (16.37 g, 86%). ^1^H-NMR (400 MHz, DMSO-d6): δ 2.49 (t, 2H), 3.61 (t, 2H), 7.03 (s, 2H), 12.37 (br, 1H). ESI *m/z* (M − H)^−^ calculated for C_7_H_7_NO_4_ 168.0; found 168.3.

*6-(2,5-Dioxo-2,5-dihydro-1H-pyrrol-1-yl)hexanoic acid* (**3b**)*.* Compound **3b** was synthesized with the experimental protocols described for **3a**. Yield was 74%, as a white solid; ^1^H-NMR (400 MHz, DMSO-d6): δ 1.24–1.17 (m, 2H), 1.51–1.44 (m, 2H), 2.17 (t, 2H), 3.39 (t, 2H), 7.01 (s, 2H), 11.98 (br, 1H). ESI *m/z* (M − H)^−^ calculated for C_7_H_7_NO_4_ 210.1; found 210.0.

*2,5-Dioxopyrrolidin-1-yl 3-(2,5-dioxo-2,5-dihydro-1H-pyrrol-1-yl)propanoate* (**4a**). A solution of **3a** (4.66 g, 22.0 mmol), 2,4,6-trimethylpyridine (11.6 mL, 88.0 mmol,) and *N*-Hydroxysuccinimide (SuOH) (5.08 g, 44.0 mmol) in THF (100 mL) was cooled to 0 °C; the trifluoroacetic anhydride (6.12 mL, 44.0 mmol) was added dropwise over 30 min. The reaction mixture was stirred for 1 h at room temperature for 1 h, and then evaporated under reduced pressure and re-dissolved in ethyl acetate (200 mL). The organic layers was washed with 1N HCl and brine, dried over anhydrous Na_2_SO_4_, and evaporated under reduced pressure to give the crude product. Purification was performed by silica column chromatography in 1:2 ethyl acetate/petroleum ether (*v/v*) to give 4.71 g (70%) of **4a** as a colorless oil, low temperature placed and converted to a white solid. ^1^H-NMR (400 MHz, DMSO-d6): δ 2.79 (s, 4H), 3.04 (t, 2H), 3.74 (t, 2H), 7.05 (s, 2H). ESI *m/z* (M + H)^+^ calculated for C_11_H_11_N_2_O_6_ 267.1; found 267.1. ESI *m/z* (M + Na)^+^ calculated for C_11_H_10_N_2_NaO_6_ 289.0; found 289.1.

*2,5-Dioxopyrrolidin-1-yl 6-(2,5-dioxo-2,5-dihydro-1H-pyrrol-1-yl)hexanoate* (**4b**)*.* Compound **4b** was synthesized with the experimental protocols described for **4a**. Yield was 69%, as a white solid; ^1^H-NMR (400 MHz, CDCl_3_-d1): δ 1.45–1.37 (m, 2H), 1.63 (m, 2H), 1.78 (m, 2H), 2.61 (t, 2H), 2.84 (s, 2H), 3.53 (t, 2H), 6.69 (s, 2H). ESI *m/z* (M + H)^+^ calculated for C_14_H_17_N_2_O_6_ 309.1; found 309.4. ESI *m/z* (M + Na)^+^ calculated for C_14_H_16_N_2_NaO_6_ 331.1; found 331.2.

*(((9H-Fluoren-9-yl)methoxy)carbonyl)-l-valyl-l-alanine* (**7a**). A solution of 5 (25.0 g, 74.0 mmol), SuOH (8.52 g, 74.0 mmol) and DCC (15.27 g, 74.0 mmol) in THF (250 mL) was stirred at room temperature for 16 h. The reaction mixture was cooled to 0 °C for 2 h, and then filtered to remove the insoluble dicyclohexylurea (DCU). The Filter cake was washed with THF; the filtrate was evaporated under reduced pressure to give the crude product as a glassy solid, which was used for the next step directly without further purification. The glassy solid was redissolved in dimethoxyethane (DME) (200 mL) and THF (100 mL), then added the aqueous sodium hydrogencarbonate solution of l-alanine (6.95 g, 78 mmol). The reaction mixture was stirred at room temperature for 16 h, poured into aqueous citric acid solution (400 mL, 15%), and filtered after the filter cake was dried under reduced pressure to give the crude product. The crude product was scattered in ether and purification was performed by ultrasound and filtration 3 times to give 16.2 g (53%) of **7a** as a white solid. ^1^H-NMR (400 MHz, DMSO-d6): δ 0.88 (dd, 6H), 1.27 (d, 3H), 1.96 (m, 1H), 3.89 (q, 1H), 4.22 (m, 4H), 7.33 (t, 2H), 7.43 (m, 3H), 7.74 (t 2H), 7.89 (d, 2H), 8.25 (d, 1H), 12.48 (s, 1H). ESI *m/z* (M + H)^+^ calculated for C_23_H_27_N_2_O_5_ 411.2; found 411.3. ESI *m/z* (M + Na)^+^ calculated for C_23_H_26_N_2_NaO_5_ 433.2; found 433.4.

*(((9H-Fluoren-9-yl)methoxy)carbonyl)-l-valyl-l-Citrulline* (**7b**). Compound **7b** was synthesized with the experimental protocols described for **7a**. Yield was 70%, as a white solid; ^1^H-NMR (400 MHz, DMSO-d6): δ 0.89 (dd, 6H), 1.42 (m, 2H), 1.58 (m, 1H), 1.72 (m, 1H), 1.98 (m, 1H), 2.96 (q, 2H), 3.94 (t, 1H), 4.16 (q, 1H), 4.25 (m,3H), 5.43 (s, 2H), 5.98 (t, 1H), 7.33 (t, 2H), 7.43 (q, 3H), 7.76 (t, 2H), 7.89 (d, 2H), 8.21 (d, 1H), 12.61 (s, 1H). ESI *m/z* (M + H)^+^ calculated for C_26_H_33_N_4_O_6_ 497.2; found 497.6. ESI *m/z* (M + Na)^+^ calculated for C_26_H_32_N_4_NaO_6_ 519.2; found 519.6.

*(9H-Fluoren-9-yl)methyl ((S)-1-(((S)-1-((4-(hydroxymethyl)phenyl)amino)-1-oxopropan-2-yl)amino)-3-methyl-1-oxobutan-2-yl)carbamate* (**8a**). p-Aminobenzyl alcohol (3.30 g, 26.8 mmol) and EEDQ (6.63 g, 26.8 mmol) were added to a solution of the **7a** (5.50 g, 13.4 mmol) in DCM (150 mL) and methanol (75 mL). The mixture was stirred at room temperature for 36 h, and the reaction mixture was evaporated under reduced pressure to give the crude product. The crude product was scattered in ether and the purification was performed by ultrasound and filtration 3 times to give 5.34 g (77%) of **8a** as a white solid. ^1^H-NMR (400 MHz, DMSO-d6): δ 0.88 (dd, 6H), 1.30 (d, 3H), 2.00 (m, 1H), 3.91 (t, 1H), 4.22 (q, 2H), 4.30 (t, 1H), 4.40 (br, 1H), 4.42 (d, 2H), 5.13 (t, 1H), 7.24 (t, 2H), 7.34 (t, 2H), 7.41 (t, 2H), 7.52 (q, 3H), 7.75 (t, 2H), 7.89 (d, 2H), 8.22 (d, 1H), 9.96 (s, 1H). ESI *m/z* (M + H)^+^ calculated for C_30_H_34_N_3_O_5_ 516.2; found 516.4. ESI *m/z* (M + Na)^+^ calculated for C_30_H_33_N_3_NaO_5_ 538.2; found 538.3.

*(9H-Fluoren-9-yl)methyl ((S)-1-(((S)-1-((4-(hydroxymethyl)phenyl)amino)-1-oxo-5-ureidopentan-2-yl)amino)-3-methyl-1-oxobutan-2-yl)carbamate* (**8b**). Compound **8b** was synthesized with the experimental protocols described for **8a**. Yield was 92%, as a light yellow solid; ^1^H-NMR (400 MHz, DMSO-d6): δ 0.86 (dd, 6H), 1.41 (m, 2H), 1.57 (m, 1H), 1.69 (m, 1H), 1.98 (m, 1H), 3.00 (m, 2H), 3.92 (q, 1H), 4.23 (q, 2H), 4.30 (q, 1H), 4.41 (q, 3H), 5.12 (t, 1H), 5.42 (s, 2H), 5.98 (t, 1H), 7.23 (d, 2H), 7.32 (m, 2H), 7.41 (m, 2H), 7.46 (d, 1H), 7.54 (d, 2H), 7.74 (t, 2H), 7.89 (d, 2H), 8.12 (d, 1H), 9.99 (s, 1H). ESI *m/z* (M + Na)^+^ calculated for C_33_H_39_N_5_NaO_6_ 624.3; found 624.5.

*(S)-2-Amino-N-((S)-1-((4-(hydroxymethyl)phenyl)amino)-1-oxopropan-2-yl)-3-methylbutanamide* (**9a**). To a solution of the **8a** (4.0 g, 7.76 mmol) in DMF (40 mL) was added piperidine (2 mL). The mixture was stirred at room temperature for 2 h, and evaporated under reduced pressure to give the crude product. Purification was performed by silica column chromatography in 20:1 DCM/methanol (*v/v*) to give 2.05 g (90%) of **9a** as a white solid. ^1^H-NMR (400 MHz, DMSO-d6): δ 0.85 (dd, 6H), 1.29 (d, 3H), 1.92 (m, 1H), 2.80 (d, 1H), 3.00 (d, 1H), 4.43 (s, 1H), 4.48 (t, 1H), 5.13 (s, 1H), 7.24 (d, 2H), 7.53 (d, 2H), 8.18 (s, 1H), 10.0 (s, 1H). ESI *m/z* (M + H)^+^ calculated for C_15_H_24_N_3_O_3_ 294.2; found 294.2. ESI *m/z* (M + Na)^+^ calculated for C_15_H_23_N_3_NaO_3_ 316.2; found 316.2.

*(S)-2-((S)-2-Amino-3-methylbutanamido)-N-(4-(hydroxymethyl)phenyl)-5-ureidopentanamide* (**9b**). Compound **9b** was synthesized with the experimental protocols described for **9a**. Yield was 84%, as a light yellow solid; ^1^H-NMR (400 MHz, DMSO-d6): δ 0.86 (dd, 6H), 1.38 (m, 2H), 1.64 (m, 2H), 1.94 (m, 1H), 2.95 (m, 2H), 3.05 (d, 1H), 4.43 (s, 2H), 4.47 (s, 1H), 5.13 (br, 1H), 5.44 (d, 2H), 6.01 (br, 1H), 7.23 (d, 2H), 7.54 (d, 2H), 8.17 (br, 1H), 10.07 (s, 1H). ESI *m/z* (M+H)^+^ calculated for C_18_H_30_N_5_O_4_ 380.2; found 380.3. ESI *m/z* (M+Na)^+^ calculated for C_18_H_29_N_5_NaO_4_ 420.2; found 402.3.

*(S)-2-(3-(2,5-Dioxo-2,5-dihydro-1H-pyrrol-1-yl)propanamido)-N-((S)-1-((4-(hydroxymethyl)phenyl)amino)-1-oxopropan-2-yl)-3-methylbutanamide* (**10a**). Compound **4a** (1.36 g, 5.11 mmol) was added to a solution of the **9a** (1.50 g, 5.11 mmol) in DMF (40 mL). The mixture was stirred at room temperature overnight, and evaporated under reduced pressure to give the crude product. The crude product was scattered in Ether and the purification was performed by ultrasound and filtration 3 times to give 1.84 g (81%) of **10a** as a light yellow solid. ^1^H-NMR (400 MHz, DMSO-d6): δ 0.83 (dd, 6H), 1.30 (d, 3H), 1.93 (m, 1H), 2.44 (t, 2H), 3.36 (m, 2H), 4.12 (q, 1H), 4.37 (m, 1H), 4.42 (d, 2H), 5.12 (t, 1H), 7.01 (s, 2H), 7.23 (d, 2H), 7.54 (d, 2H), 8.05 (d, 1H), 8.19 (d, 1H), 9.83 (s, 1H). ESI *m/z* (M + H)^+^ calculated for C_22_H_29_N_4_O_6_ 445.2; found 445.7. ESI *m/z* (M + Na)^+^ calculated for C_22_H_28_N_4_NaO_6_ 467.2; found 467.4.

*6-(2,5-Dioxo-2,5-dihydro-1H-pyrrol-1-yl)-N-((S)-1-(((S)-1-((4-(hydroxymethyl)phenyl)amino)-1-oxopropan-2-yl)amino)-3-methyl-1-oxobutan-2-yl)hexanamide* (**10b**). Compound **10b** was synthesized with the experimental protocols described for **10a**. Yield was 87%, as a light yellow solid; ^1^H-NMR (400 MHz, DMSO-d6): δ 0.84 (dd, 6H), 1.17 (m, 2H), 1.30 (d, 3H), 1.47 (m, 4H), 1.96 (m, 1H), 2.14 (m, 2H), 3.38 ( t, 2H), 4.16 (q, 1H), 4.37 (m, 1H), 4.42 (d, 2H), 5.11 (t, 1H), 7.01 (s, 2H), 7.22 (d, 2H), 7.53 (d, 2H), 7.83 (d, 1H), 8.16 (d, 1H), 9.87 (s, 1H). ESI *m/z* (M + H)^+^ calculated for C_25_H_35_N_4_O_6_ 487.3; found 487.5. ESI *m/z* (M + Na)^+^ calculated for C_25_H_34_N_4_NaO_6_ 509.2; found 509.4.

*6-(2,5-Dioxo-2,5-dihydro-1H-pyrrol-1-yl)-N-((S)-1-(((S)-1-((4-(hydroxymethyl)phenyl)amino)-1-oxo-5-ureidopentan-2-yl)amino)-3-methyl-1-oxobutan-2-yl)hexanamide* (**10c**). Compound **10c** was synthesized with the experimental protocols described for **10a**. Yield was 79%, as a light yellow solid; ^1^H-NMR (400 MHz, DMSO-d6): δ 0.83 (dd, 6H), 1.20 (m, 4H), 1.47 (m, 4H), 1.58 (m, 1H), 1.68 (m, 1H), 1.95 (m, 1H), 2.18 (m, 2H), 2.98 (m, 2H), 3.32 (t, 2H), 4.19 (t, 1H), 4.36 (m, 1H), 4.42 (d, 2H), 5.12 (t, 1H), 5.43 (s, 2H), 5.99 (t, 1H), 7.01 (s, 2H), 7.22 (d, 2H), 7.54 (d, 2H), 7.83 (d, 1H), 8.09 (d, 1H), 9.93 (s, 1H). ESI *m/z* (M + H)^+^ calculated for C_28_H_41_N_6_O_7_ 573.3; found 573.6. ESI *m/z* (M + Na)^+^ calculated for C_25_H_34_N_4_NaO_6_ 595.3; found 595.7.

*4-((S)-2-((S)-2-(3-(2,5-Dioxo-2,5-dihydro-1H-pyrrol-1-yl)propanamido)-3-methylbutanamido)propanamido)benzyl (4-nitrophenyl) carbonate* (**11a**). Bis(4-Nitrophenyl) Carbonate (0.57 g, 1.89 mmol) and DIPEA (250 μL, 1.42 mmol) were added to a solution of the **10a** (0.42 g, 0.95 mmol) in DMF (15 mL). The mixture was stirred at rt for 5 h, and evaporated under reduced pressure to give the crude product. The crude product was scattered in ether and the purification was performed by ultrasound and filtration 3 times to give 0.5 g (86%) of **11a** as a light yellow solid. ^1^H-NMR (400 MHz, DMSO-d6): δ 0.84 (dd, 6H), 1.31 (d, 3H), 1.94 (m, 1H), 2.45 (t, 2H), 3.60 (m, 2H), 4.13 (q, 1H), 4.38 (m, 1H), 5.24 (s, 2H), 7.00 (s, 2H), 7.41 (d, 2H), 7.57 (dt, 2H), 7.64 (d, 2H), 8.02 (d, 1H), 8.19 (d, 1H), 8.31 (dt, 2H), 9.96 (s, 1H). ESI *m/z* (M + H)^+^ calculated for C_29_H_32_N_5_O_10_ 610.2; found 610.4. ESI *m/z* (M + Na)^+^ calculated for C_29_H_31_N_5_NaO_10_ 632.2; found 632.6.

*4-((S)-2-((S)-2-(6-(2,5-Dioxo-2,5-dihydro-1H-pyrrol-1-yl)hexanamido)-3-methylbutanamido)propanamido)benzyl (4-nitrophenyl) carbonate* (**11b**). Compound **11b** was synthesized with the experimental protocols described for **11a**. Yield was 93%, as a light yellow solid; ^1^H-NMR (400 MHz, DMSO-d6): δ 0.85 (dd, 6H), 1.18 (m, 2H), 1.31 (d, 3H), 1.47 (m, 4H), 1.96 (m, 1H), 2.15 (m, 2H), 3.36 ( t, 2H), 4.17 (q, 1H), 4.39 (m, 1H), 5.24 (s, 2H), 7.00 (s, 2H), 7.41 (d, 2H), 7.56 (dt, 2H), 7.64 (d, 2H), 7.80 (d, 1H), 8.17 (d, 1H), 8.31 (dt, 2H), 10.00 (s, 1H). ESI *m/z* (M + H)^+^ calculated for C_32_H_38_N_5_O_10_ 652.3; found 652.6. ESI *m/z* (M + Na)^+^ calculated for C_32_H_37_N_5_NaO_10_ 674.2; found 674.6.

*4-((S)-2-((S)-2-(6-(2,5-Dioxo-2,5-dihydro-1H-pyrrol-1-yl)hexanamido)-3-methylbutanamido)-5-ureidopentanamido)benzyl (4-nitrophenyl) carbonate* (**11c**). Compound **11c** was synthesized with the experimental protocols described for **11a**. Yield was 92%, as a light yellow solid; ^1^H-NMR (400 MHz, DMSO-d6): δ 0.84 (dd, 6H), 1.22 (m, 4H), 1.47 (m, 4H), 1.58 (m, 1H), 1.68 (m, 1H), 1.95 (m, 1H), 2.17 (m, 2H), 3.00 (m, 2H), 3.28 (t, 2H), 4.19 (t, 1H), 4.38 (m, 1H), 5.24 (s, 2H), 5.44 (s, 2H), 6.01 (t, 1H), 7.01 (s, 2H), 7.41 (d, 2H), 7.56 (dt, 2H), 7.66 (d, 2H), 7.83 (d, 1H), 8.15 (d, 1H), 8.32 (dt, 2H), 10.10 (s, 1H). ESI *m/z* (M + H)^+^ calculated for C_35_H_44_N_7_O_11_ 738.3; found 738.6. ESI *m/z* (M + Na)^+^ calculated for C_35_H_43_N_7_NaO_11_ 760.3; found 760.6.

*4-((S)-2-((S)-2-(3-(2,5-Dioxo-2,5-dihydro-1H-pyrrol-1-yl)propanamido)-3-methylbutanamido)propanamido)benzyl((S)-1-(((S)-1-(((3R,4S,5S)-1-((S)-2-((1R,2R)-3-(((1S,2R)-1-hydroxy-1-phenylpropan-2-yl)amino)-1-methoxy-2-methyl-3-oxopropyl)pyrrolidin-1-yl)-3-methoxy-5-methyl-1-oxoheptan-4-yl)(methyl)amino)-3-methyl-1-oxobutan-2-yl)amino)-3-methyl-1-oxobutan-2-yl)(methyl)carbamate* (**12a**). To a solution of the **11a** (50.94 mg, 0.0836 mmol), MMAE (40 mg, 0.0557 mmol), HOBt (7.53 mg, 0.0557 mmol) in DMF (3 mL) was added DIPEA (20 μL, 0.1114 mmol). The mixture was stirred at rt overnight, and then poured into water (20 mL). The mixture was extracted with ethyl acetate (3 × 20 mL), the organic layers were combined, washed with brine, dried over anhydrous Na_2_SO_4_, and evaporated under reduced pressure to give the crude product. Purification was performed by silica column chromatography in 50:1-10:1 DCM/methanol (*v/v*) to give 47.6 mg (72%) of **12a** as a white solid. ^1^H-NMR (400 MHz, DMSO-d6): δ 0.88–0.75 (m, 23H), 1.05–0.97 (m, 6H), 1.30–1.23 (m, 6H), 1.54–1.48 (m, 2H), 1.83–1.70 (m, 3H), 1.95–1.91 (m, 2H), 2.14–2.08 (m, 2H), 2.28–2.22 (m, 1H), 2.48–2.38 (m, 4H), 2.89–2.83 (dd, 3H), 2.97 (s, 1H), 3.24–3.12 (m, 8H), 3.33–3.30 (m, 2H), 3.47 (br, 1H), 3.64–3.55 (m, 3H), 4.05–3.92 (m, 2H), 4.13 (t, 1H), 4.26 (t, 1H), 4.49–4.35 (m, 3H), 4.76–4.62 (m, 1H), 5.09–4.96 (m, 2H), 5.44–5.36 (d, 1H), 7.01 (s, 2H), 7.16 (m, 1H), 7.35–7.26 (m, 6H), 7.57 (d, 2H), 7.66 (d, 0.5H), 7.91 (d, 0.5H), 8.05 (d, 1H), 8.12 (d, 0.5H), 8.22 (d, 1H), 8.34 (br, 0.5H), 9.92 (d, 1H). ESI *m/z* (M + H)^+^ calculated for C_62_H_94_N_9_O_14_ 1188.6920; found 1188.6913. ESI *m/z* (M + Na)^+^ calculated for C_62_H_93_N_9_NaO_14_ 1210.6740; found 1210.6734.

*4-((S)-2-((S)-2-(6-(2,5-Dioxo-2,5-dihydro-1H-pyrrol-1-yl)hexanamido)-3-methylbutanamido)propanamido)benzyl((S)-1-(((S)-1-(((3R,4S,5S)-1-((S)-2-((1R,2R)-3-(((1S,2R)-1-hydroxy-1-phenylpropan-2-yl)amino)-1-methoxy-2-methyl-3-oxopropyl)pyrrolidin-1-yl)-3-methoxy-5-methyl-1-oxoheptan-4-yl)(methyl)amino)-3-methyl-1-oxobutan-2-yl)amino)-3-methyl-1-oxobutan-2-yl)(methyl)carbamate* (**12b**). Compound **12b** was synthesized with the experimental protocols described for 12a. Yield was 80%, as a white solid; ^1^H-NMR (400 MHz, DMSO-d6): δ 0.88–0.73 (m, 25H), 1.05–0.93 (m, 6H), 1.30–1.21 (m, 6H), 1.55–1.43 (m, 6H), 1.81–1.71 (m, 3H), 2.00–1.91 (m, 2H), 2.19–2.06 (m, 4H), 2.28–2.21 (m, 1H), 2.43–2.39 (d, 1H), 2.88–2.83 (dd, 3H), 2.97 (s, 1H), 3.24–3.12 (m, 8H), 3.33–3.30 (m, 2H), 3.49–3.38 (m, 3H), 3.59–3.53 (m, 1H), 4.01 (m, 2H), 4.17 (t, 1H), 4.26 (t, 1H), 4.49–4.36 (m, 3H), 4.75–4.61 (m, 1H), 5.11–4.96 (m, 2H), 5.43–5.36 (dd, 1H), 7.01 (s, 2H), 7.16 (m, 1H), 7.34–7.24 (m, 6H), 7.57 (d, 2H), 7.66 (d, 0.5H), 7.83 (d, 1H), 7.91 (d, 0.5H), 8.10 (d, 0.5H), 8.19 (d, 1H), 8.35 (d, 0.5H), 9.95 (d, 1H). ESI *m/z* (M + H)^+^ calculated for C_65_H_100_N_9_O_14_ 1230.7390; found 1230.7380. ESI *m/z* (M + Na)^+^ calculated for C_65_H_99_N_9_NaO_14_ 1252.7209; found 1252.7202.

*4-((S)-2-((S)-2-(6-(2,5-Dioxo-2,5-dihydro-1H-pyrrol-1-yl)hexanamido)-3-methylbutanamido)-5-ureidopentanamido)benzyl((S)-1-(((S)-1-(((3R,4S,5S)-1-((S)-2-((1R,2R)-3-(((1S,2R)-1-hydroxy-1-phenylpropan-2-yl)amino)-1-methoxy-2-methyl-3-oxopropyl)pyrrolidin-1-yl)-3-methoxy-5-methyl-1-oxoheptan-4-yl)(methyl)amino)-3-methyl-1-oxobutan-2-yl)amino)-3-methyl-1-oxobutan-2-yl)(methyl)carbamate* (**12c**). Compound **12c** was synthesized with the experimental protocols described for **12a**. Yield was 64%, as a white solid; ^1^H-NMR (400 MHz, DMSO-d6): δ 0.88–0.73 (m, 25H), 1.07–0.97 (m, 6H), 1.59–1.15 (m, 14H), 1.80–1.66 (m, 3H), 1.99–1.93 (m, 2H), 2.20–2.07 (m, 3H), 2.27 (d, 1H), 2.41 (d, 1H), 2.86 (dd, 3H), 3.03–2.94 (m, 3H), 3.32–3.12 (m, 11H), 3.36 (t, 2H), 3.56 (m, 1H), 4.01 (m, 2H), 4.18 (t, 1H), 4.26 (t, 1H), 4.49–4.34 (m, 3H), 4.75–4.62 (m, 1H), 5.11–4.95 (m, 2H), 5.60–5.20 (m, 3H), 6.01 (br, 1H), 7.01 (s, 2H), 7.17 (br, 1H), 7.34–7.24 (m, 6H), 7.41 (br, 0.5H), 7.58 (d, 2H), 7.66 (d, 0.5H), 7.82 (d, 1H), 7.92 (d, 0.5H), 8.12 (d, 1H), 8.35 (br, 0.5H), 10.00 (d, 1H). ESI *m/z* (M + H)^+^ calculated for C_68_H_106_N_11_O_15_ 1316.7870; found 1316.7864. ESI *m/z* (M + Na)^+^ calculated for C_68_H_105_N_11_NaO_15_ 1338.7689; found 1338.7678.

Mass spectrometry and NMR details of the above synthesized compounds are provided in the Supplementary Materials Figure S1–S38.

## 5. Conclusions

Currently, ADC is a promising and rapidly growing area of targeted therapeutics for oncology; however, one of the biggest challenges in their development has been the generation of suitable linkers for the conjugation of antibody and cytotoxins. ADCs that load the popular MMAE usually contain VC-based linkers. In this study, a novel ADC that conjugated MMAE to a humanized anti-HER2 antibody via a VA-based cleavable linker was generated to explore its characteristics when used as an anti-tumor drug.

First, VA-containing drug payload exhibits similar performance as VC in terms of in vitro stability and enzymatic activity, and VA-based ADC may be preferred to VC-based ADC on the conjugate process and aggregation. Further, we observed that the VA-based ADC displays favorable potency in vitro and in vivo while not producing significant systemic toxicity during the treatment and recovery period in xenograft studies. In summary, VA-based ADC with MMAE as the payload is shown to be efficacious in the model of HER2-overexpressing cancer, and may have advantages in the generation process and material source of the product compared to current linker technology.

## Figures and Tables

**Table 1:** Cytotoxicity of ADCs, antibodies and MMAE in cancer cell lines in vitro.

Cell Line	HER2 Status	IC_50_ (nM)					
		mil40-12a	mil40-12b	mil40-12c	mil40	Herceptin	MMAE
BT-474	HER2+	0.09	0.08	0.08	1.54	1.75	0.30
SK-BR-3	HER2+	0.02	0.02	0.02	0.27	0.19	0.12
NCI-N87	HER2+	0.05	0.06	0.05	0.36	0.43	0.16
SK-OV-3	HER2+	0.22	0.15	0.16	2762.61	>2703	0.64
MCF-7	HER2−	232.47	166.71	721.79	>3438	>2703	0.22
MDA-MB-468	HER2−	523.12	468.85	606.28	552.55	1558.71	0.07

**Table 2:** The *p* value in the unpaired *t*-test between the vehicle and ADC treatment groups.

Groups	RBC-1	RBC-2	HGB-1	HGB-2	WBC-1	WBC-2	PLT-1	PLT-2	Neut-1	Neut-2	Lymph-1	Lymph-2
vehicle vs. 1.0 mpk	0.6519	0.3101	0.8766	0.5107	0.6839	0.3233	0.5315	0.8652	0.8376	0.2773	0.8002	0.6116
vehicle vs. 2.5 mpk	0.3300	0.4041	0.1449	0.6756	0.2319	0.7879	0.2542	0.8389	0.6110	0.5141	0.2302	0.5083
vehicle vs. 5.0 mpk	0.9090	0.4871	1.0000	0.6930	0.5247	0.1460	0.2221	0.4394	0.2217	0.5862	0.3430	0.4378

## Supplementary Materials

Supplementary materials can be found at www.mdpi.com/1422-0067/18/9/1860/s1.

## Abbreviations

ADC	Antibody-drug conjugate
DAR	Drug to antibody ratio
HER2	Human epithelial growth factor receptor 2
HIC	Hydrophobic interaction chromatography
HPLC	High performance liquid chromatographic
MMAE	Auristatin E
MS	Mass spectrometric
NAC	*N*-Acetyl-l-cysteine
NMR	Nuclear magnetic resonance
SEC	Size exclusion chromatography
TCEP	*tris*(2-Carboxyethyl)phosphine hydrochloride
VA	Valine-alanine
VC	Valine-citrulline
RBC	Red blood cell
HGB	Hemoglobin
WBC	White blood cell
PLT	Platelet
Neut	Neutrophil
Lymph	Lymphocyte
