# Supplementary material for: Development and Properties of Valine-Alanine based Antibody-Drug Conjugates with Monomethyl Auristatin E as the Potent Payload
Source: Int J Mol Sci. 2017 Aug 25;18(9):1860. doi: 10.3390/ijms18091860 (PMC5618509; doi:10.3390/ijms18091860)
Supplement: Supplementary file 1 [file ijms-18-01860-s001.pdf]

Supplementary Materials :

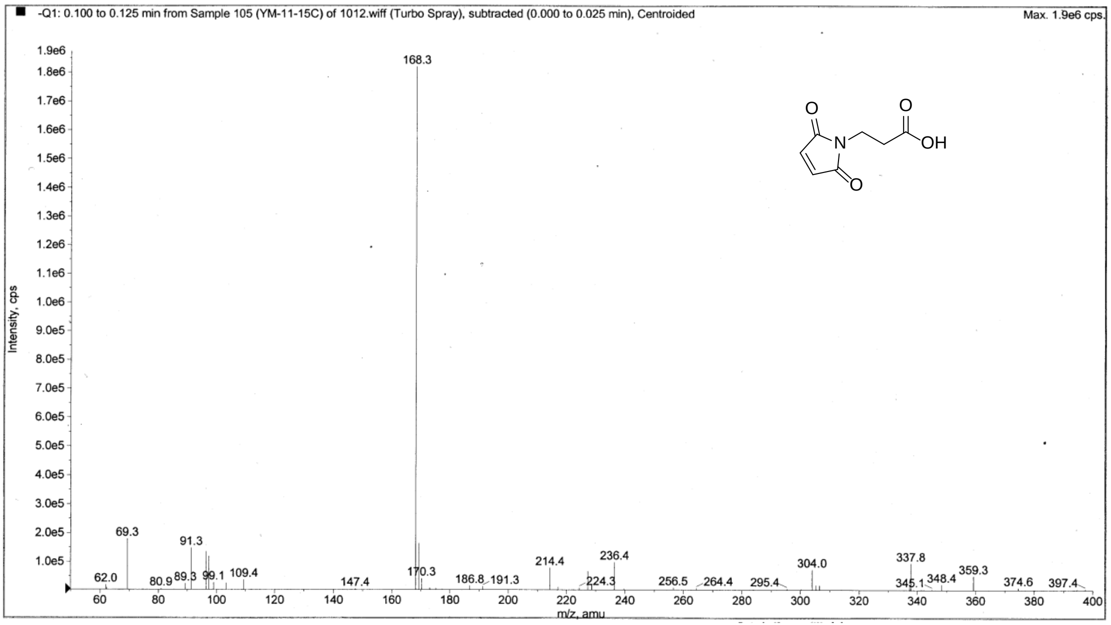

Figure S1. The ESI-MS spectrum of compound *3a*.

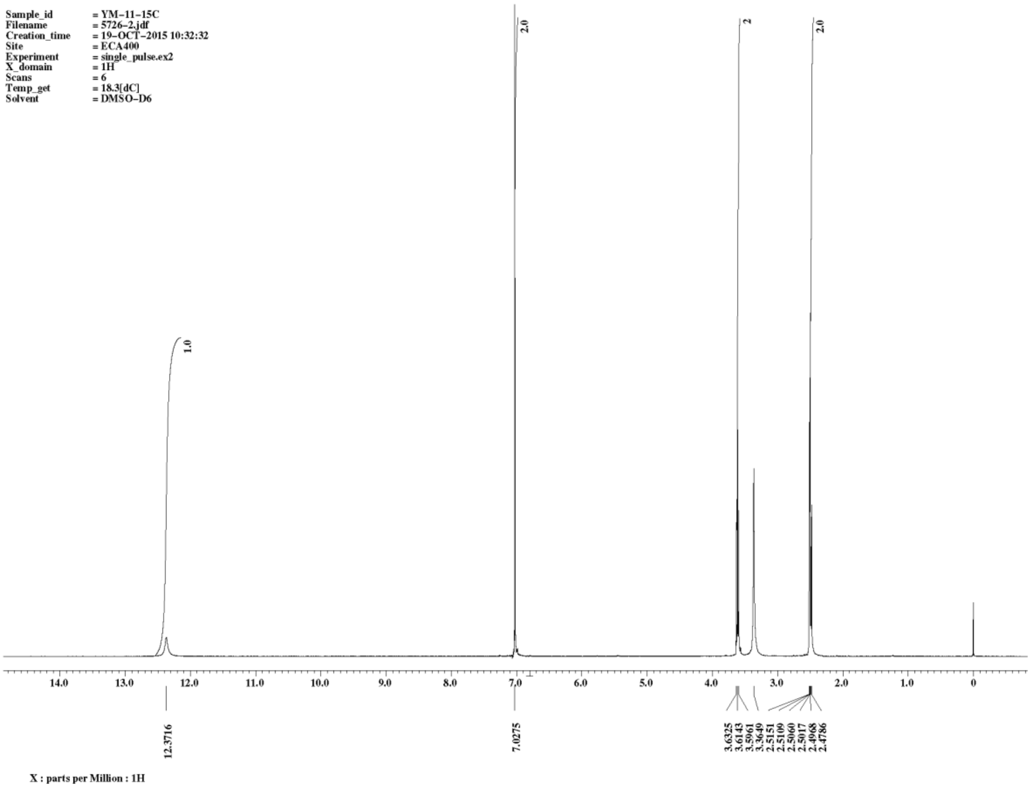

Figure S2. The ^{1}H-NMR spectrum of compound *3a*.

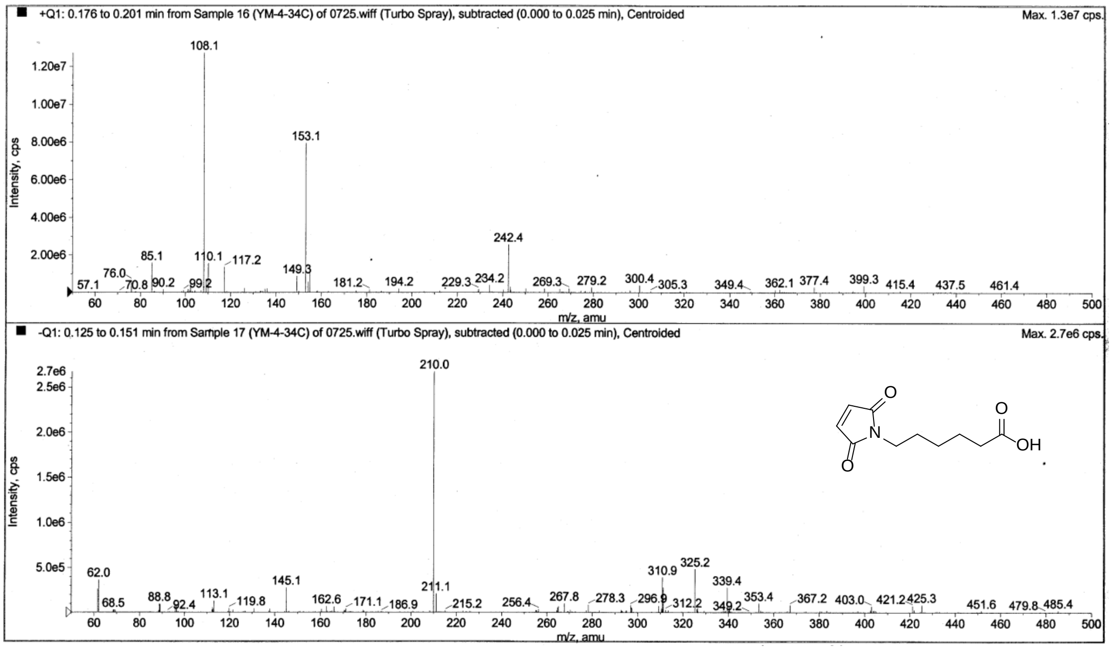

Figure S3. The ESI-MS spectrum of compound *3b*.

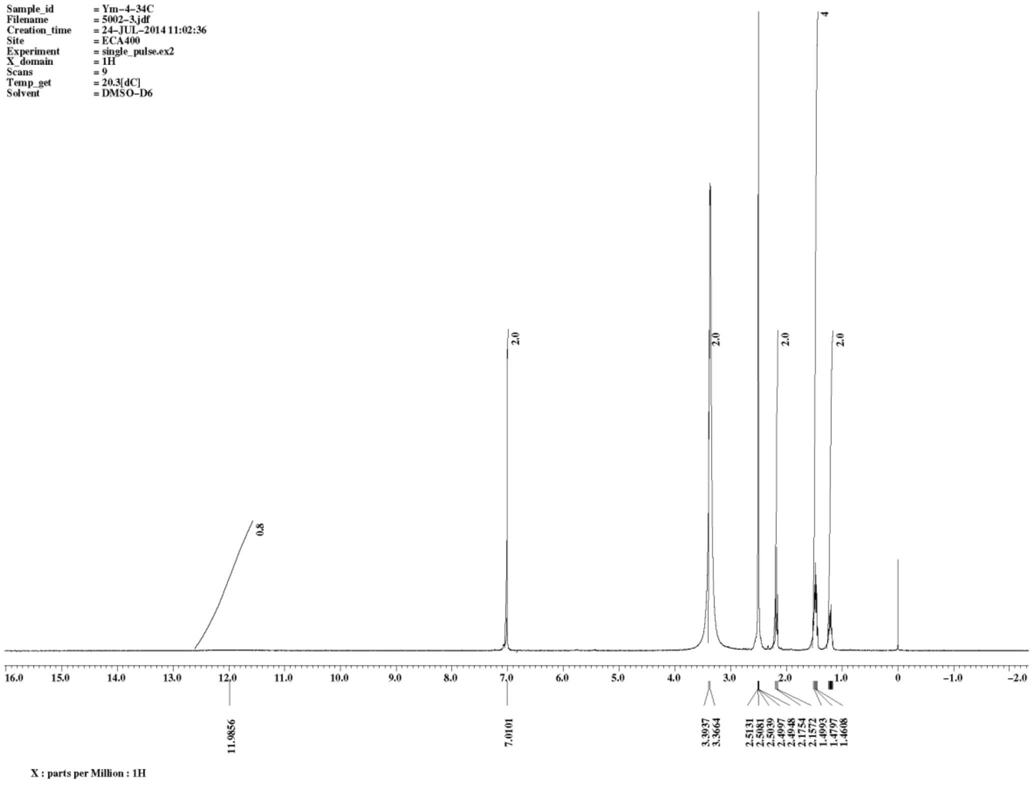

Figure S4. The ^{1}H-NMR spectrum of compound *3b*.

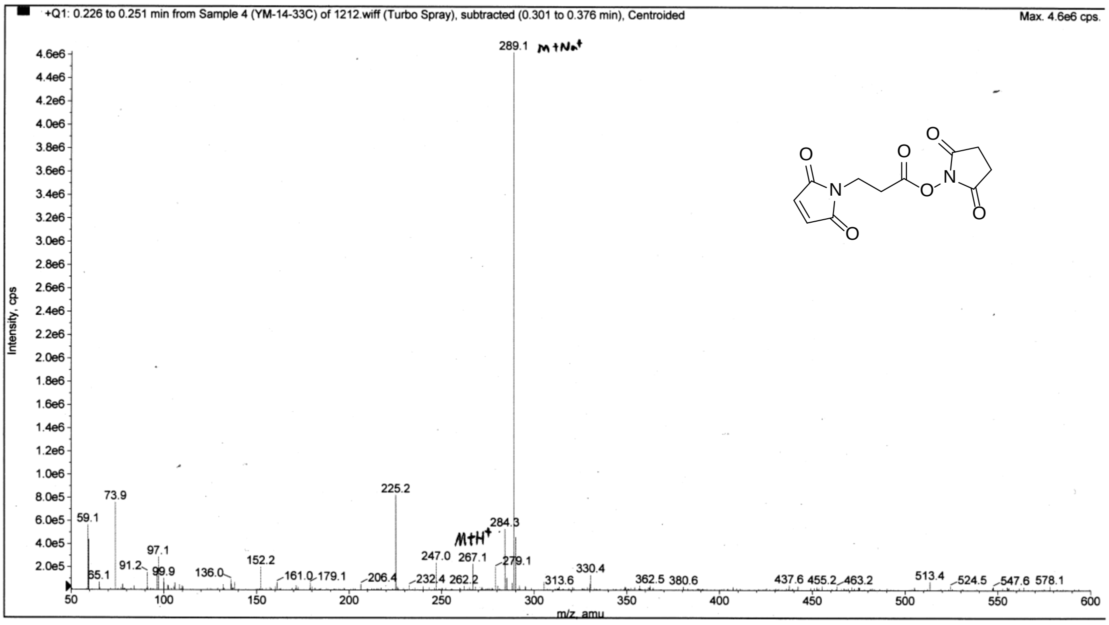

Figure S5. The ESI-MS spectrum of compound *4a*.

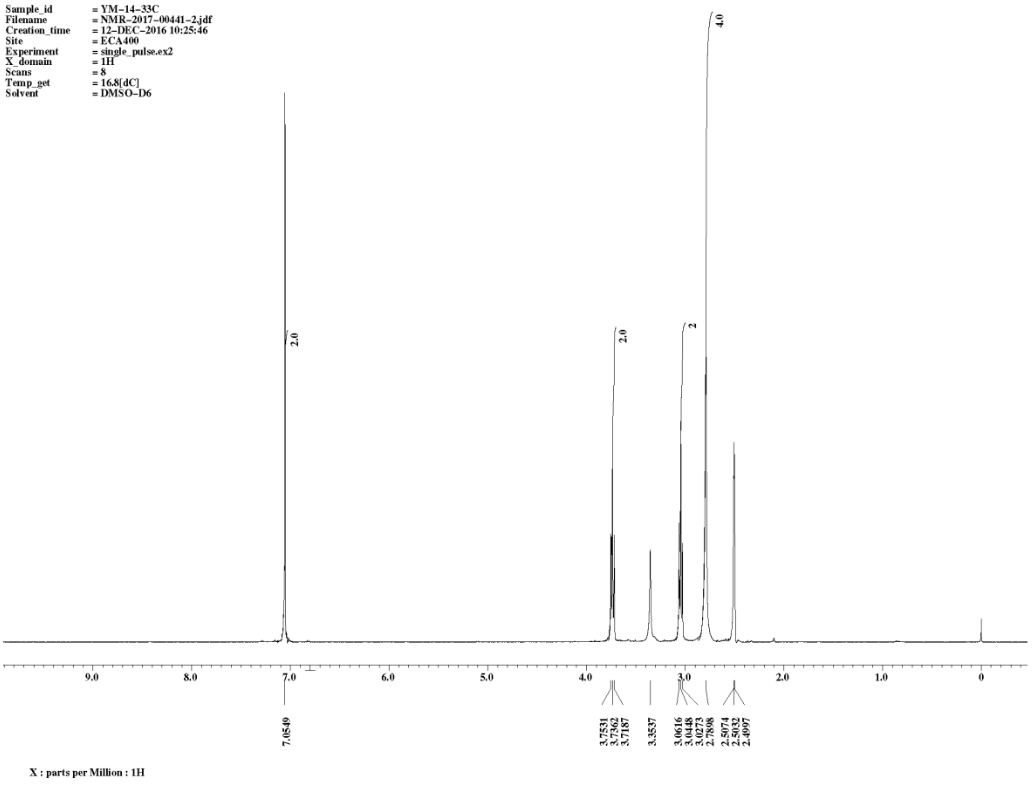

Figure S6. The ^{1}H-NMR spectrum of compound *4a*.

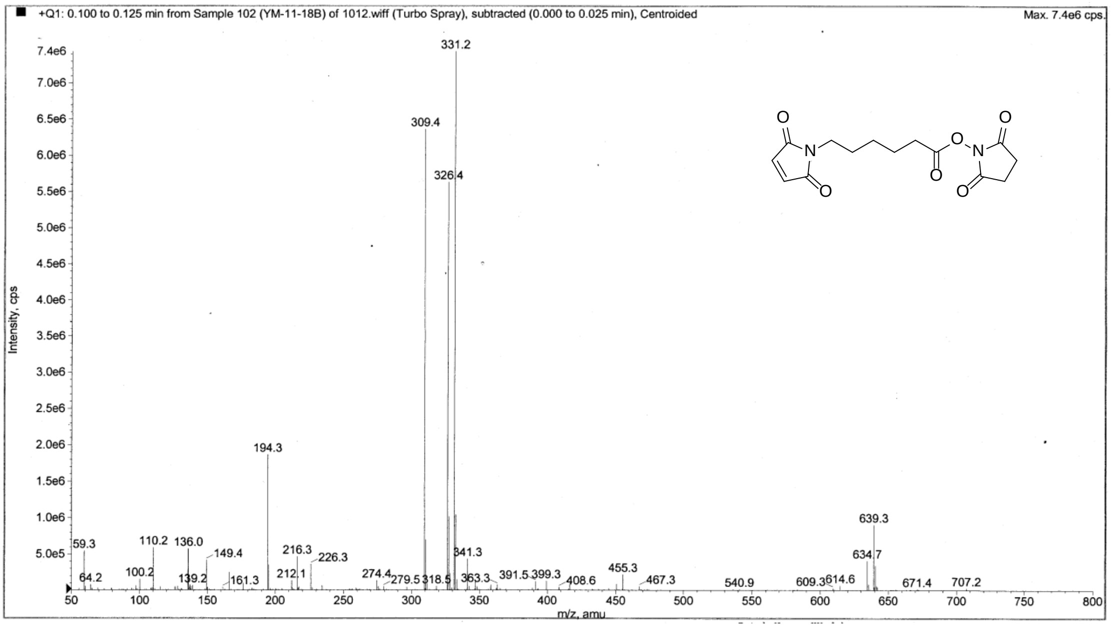

Figure S7. The ESI-MS spectrum of compound *4b*.

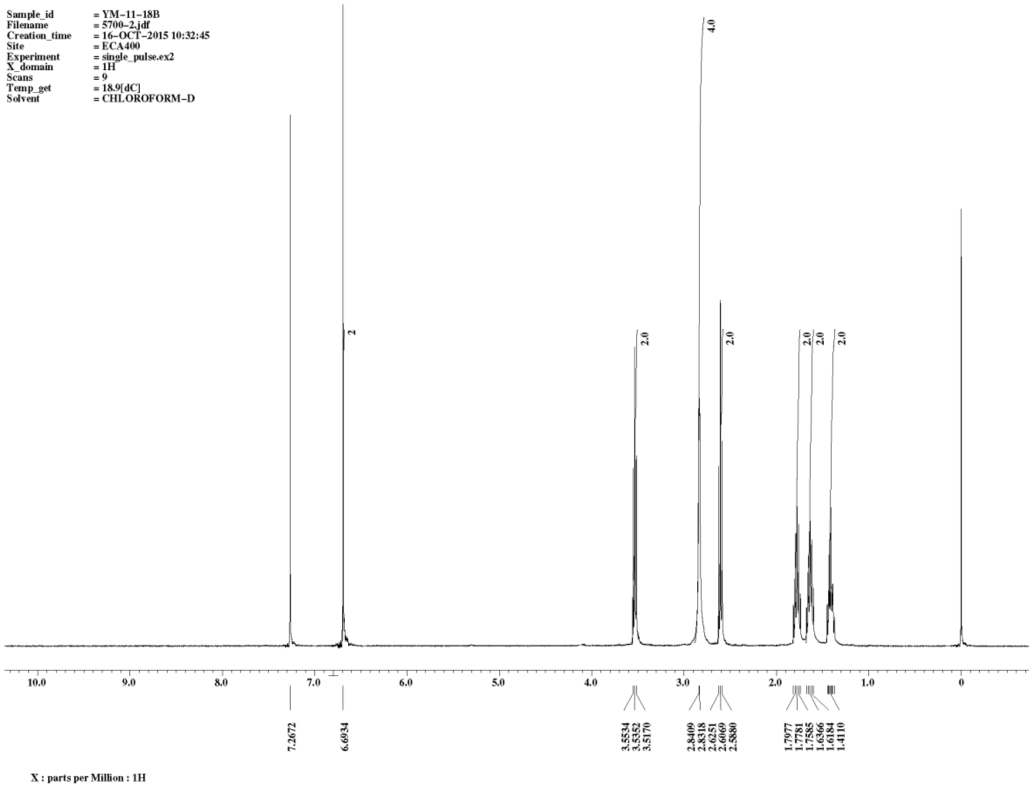

Figure S8. The ^{1}H-NMR spectrum of compound *4b*.

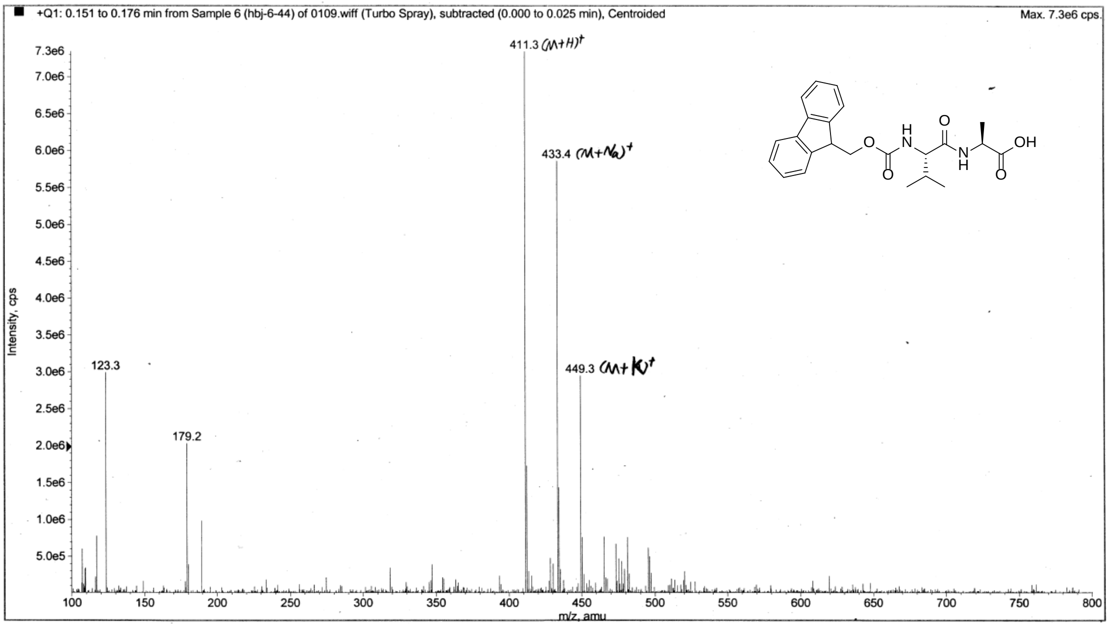

Figure S9. The ESI-MS spectrum of compound *7a*.

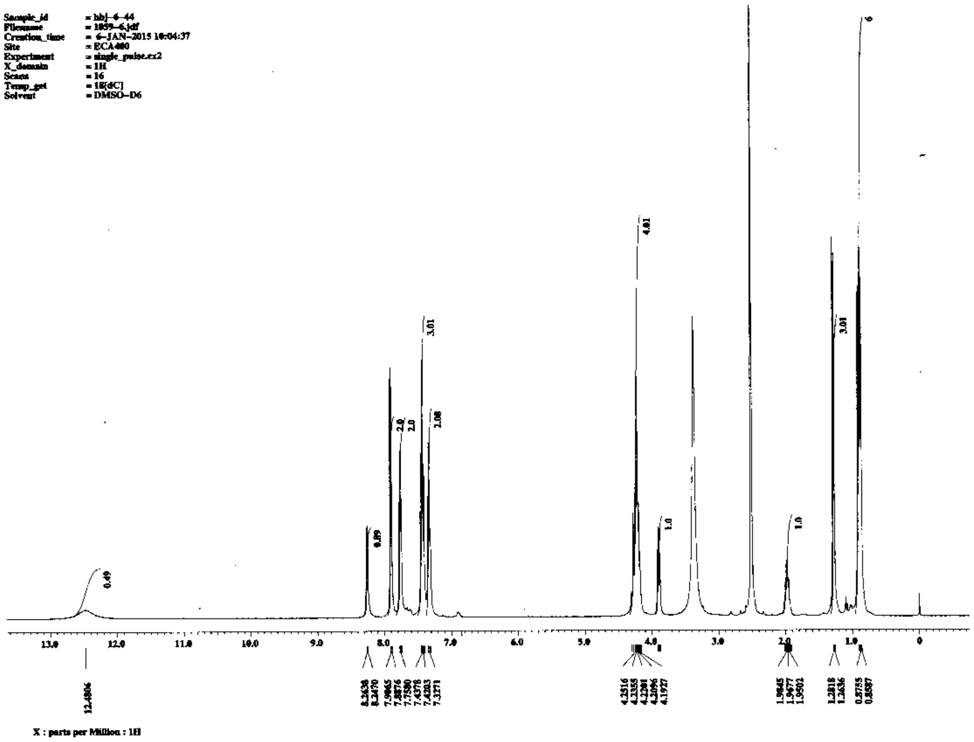

Figure S10. The ^{1}H-NMR spectrum of compound *7a*.

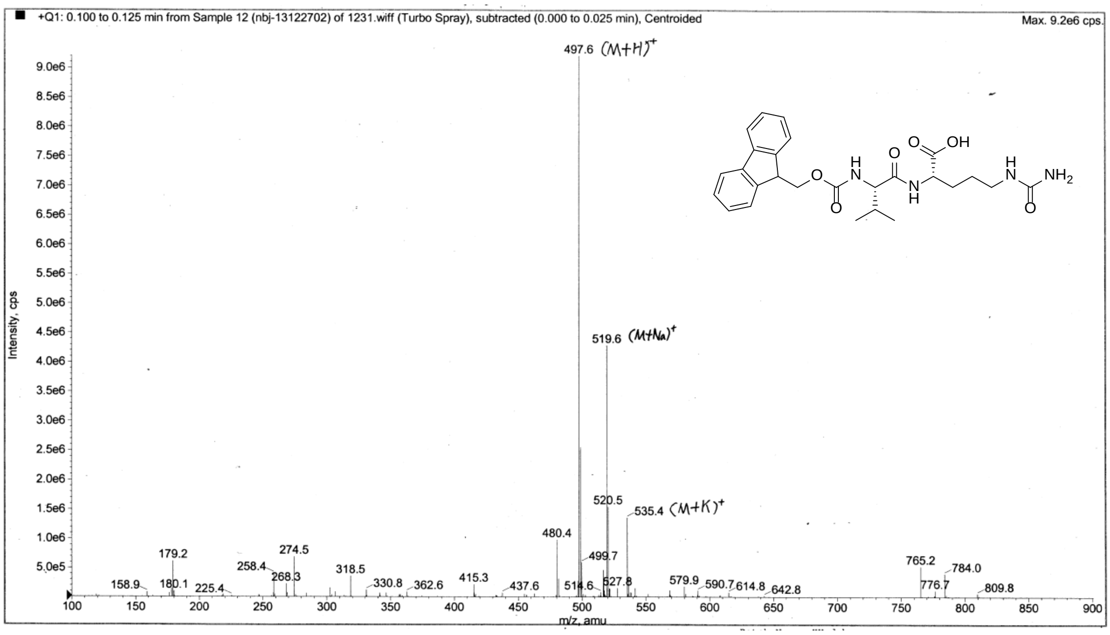

Figure S11. The ESI-MS spectrum of compound *7b*.

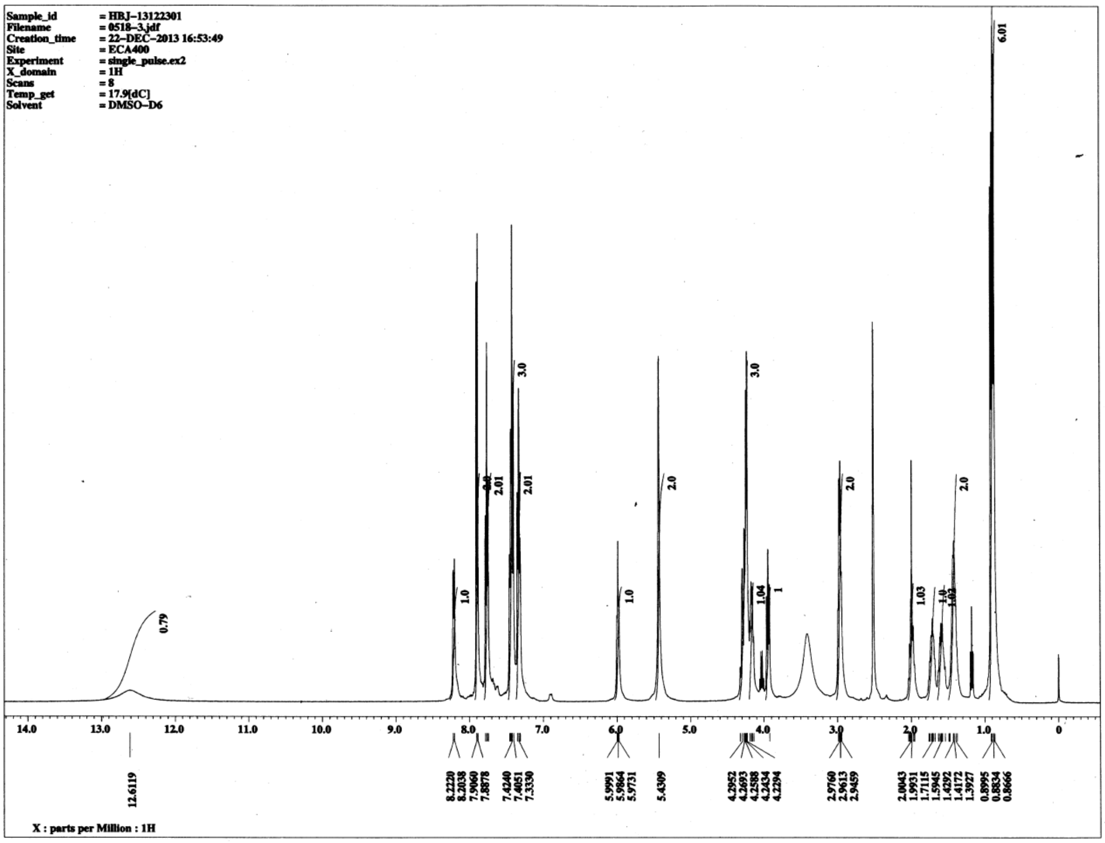

Figure S12. The ^{1}H-NMR spectrum of compound *7b*.

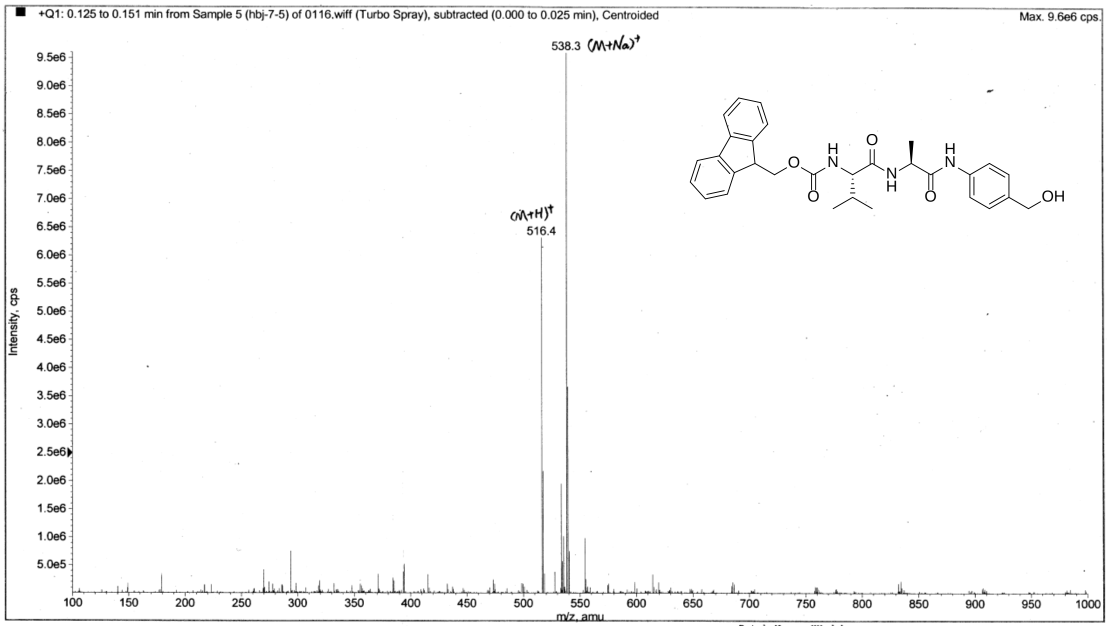

Figure S13. The ESI-MS spectrum of compound *8a*.

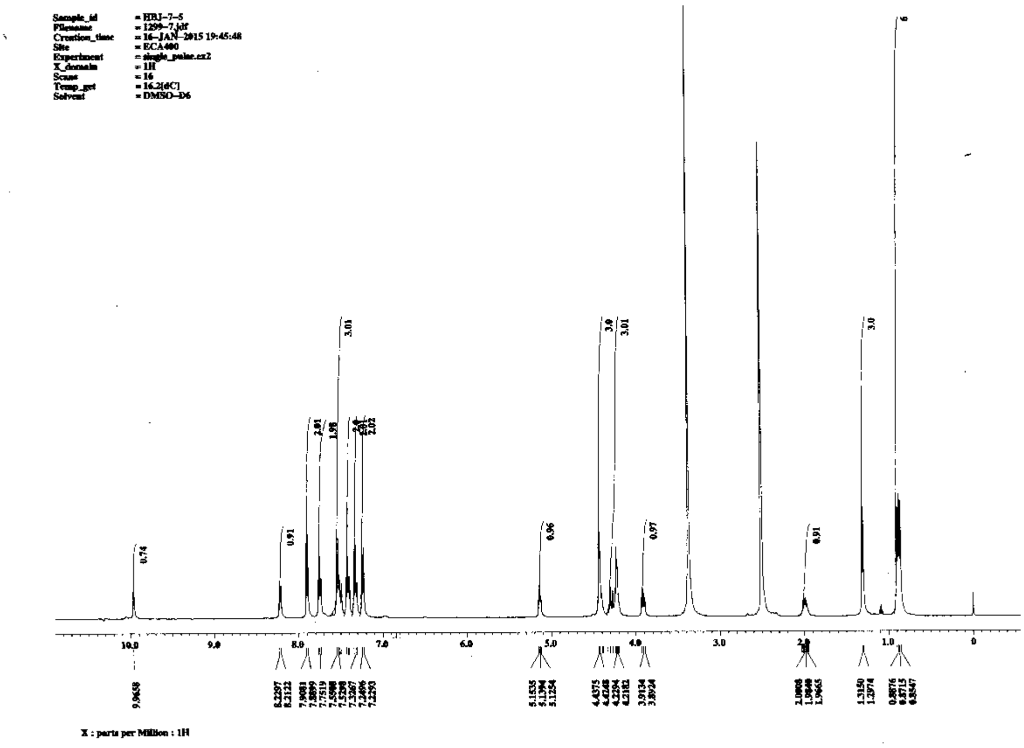

Figure S14. The ^{1}H-NMR spectrum of compound *8a*.

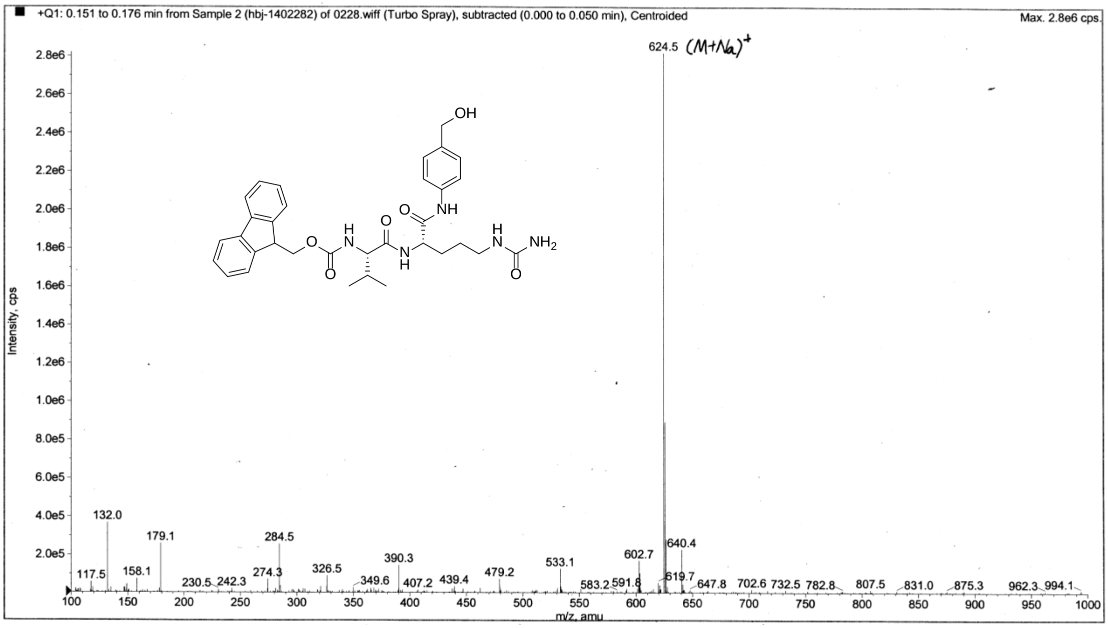

Figure S15. The ESI-MS spectrum of compound *8b*.

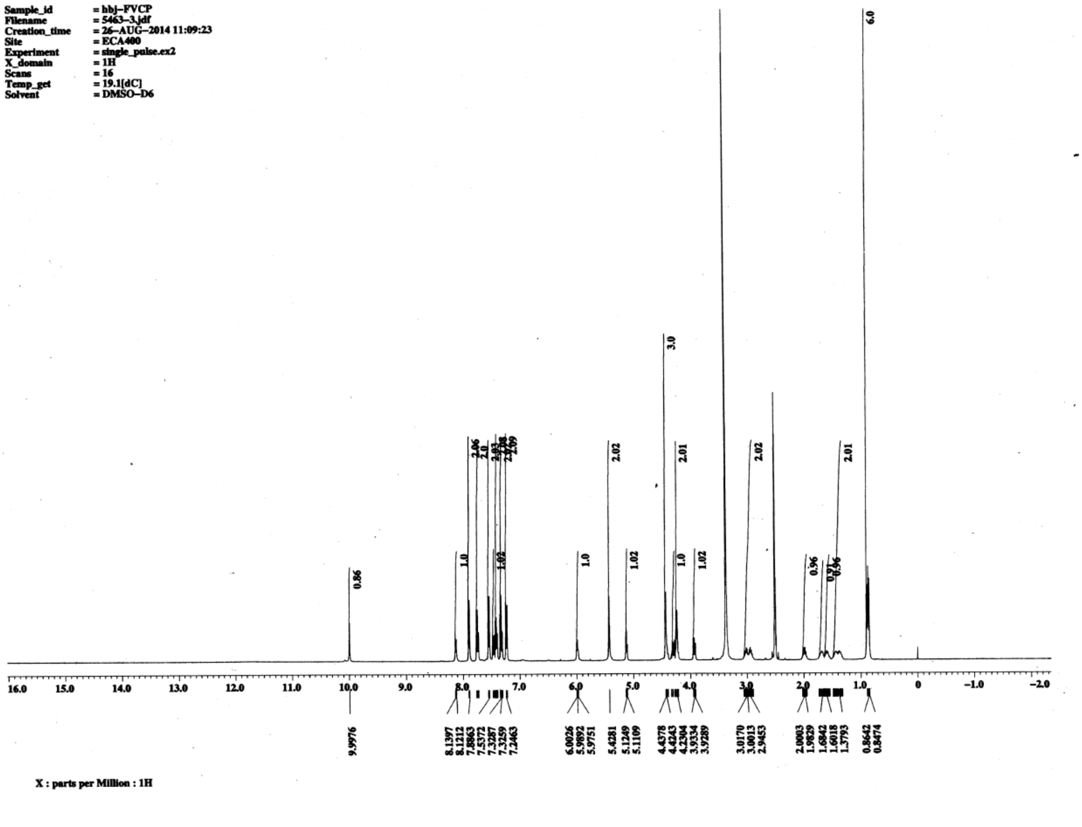

Figure S16. The ^{1}H-NMR spectrum of compound *8b*.

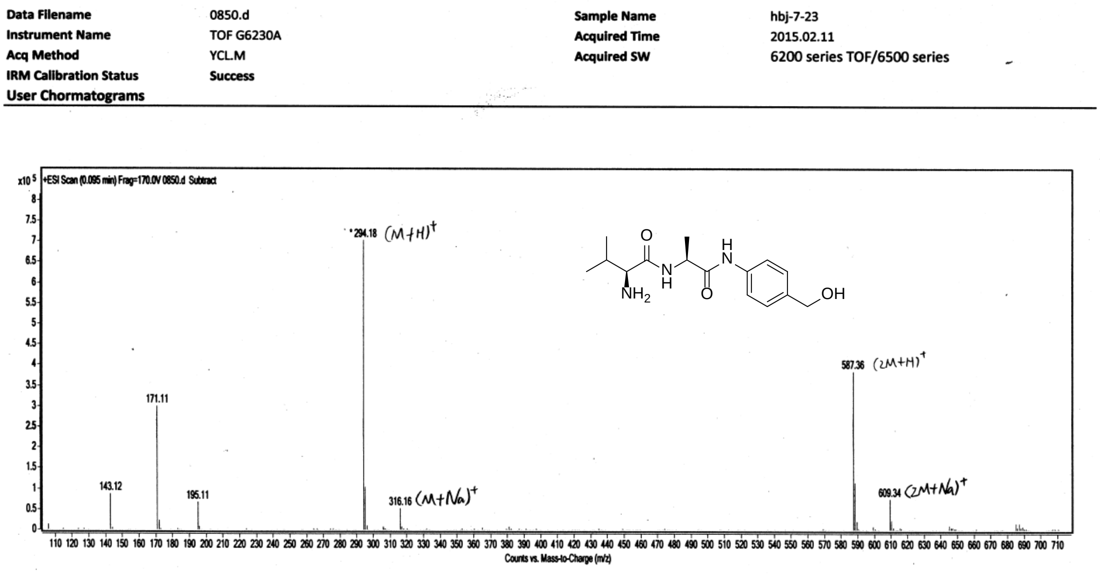

Figure S17. The ESI-MS spectrum of compound *9a*.

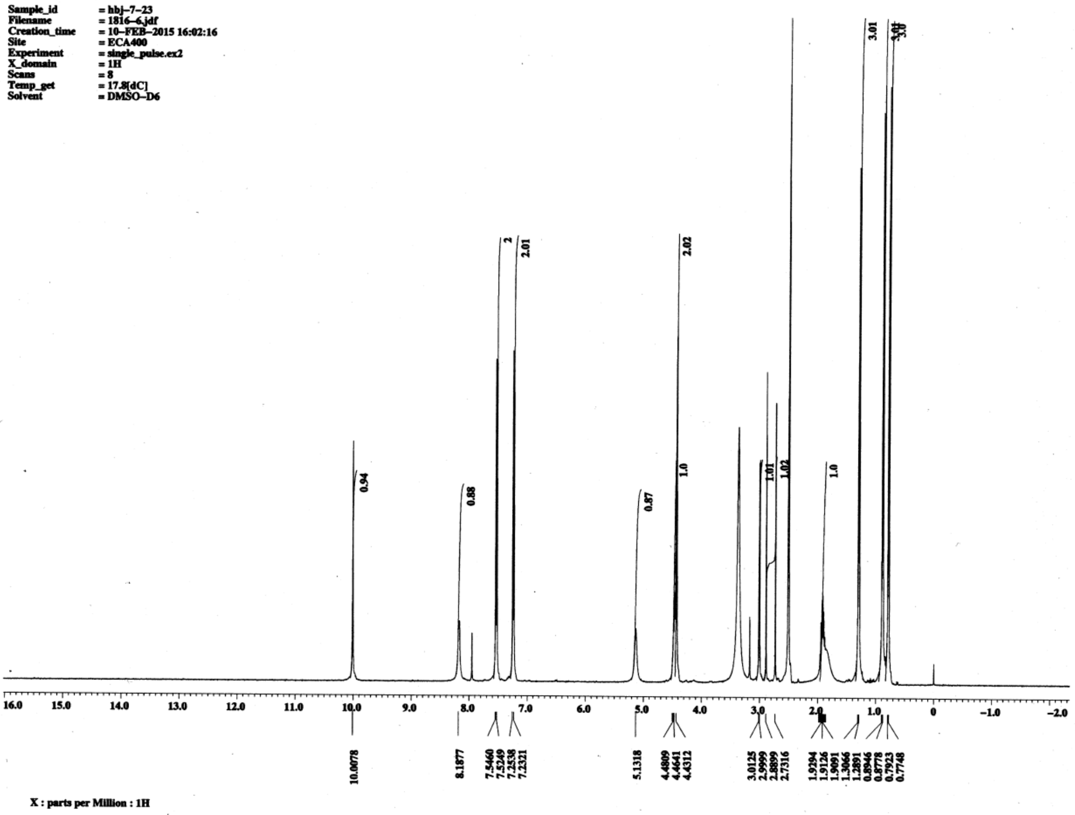

Figure S18. The ^{1}H-NMR spectrum of compound *9a*.

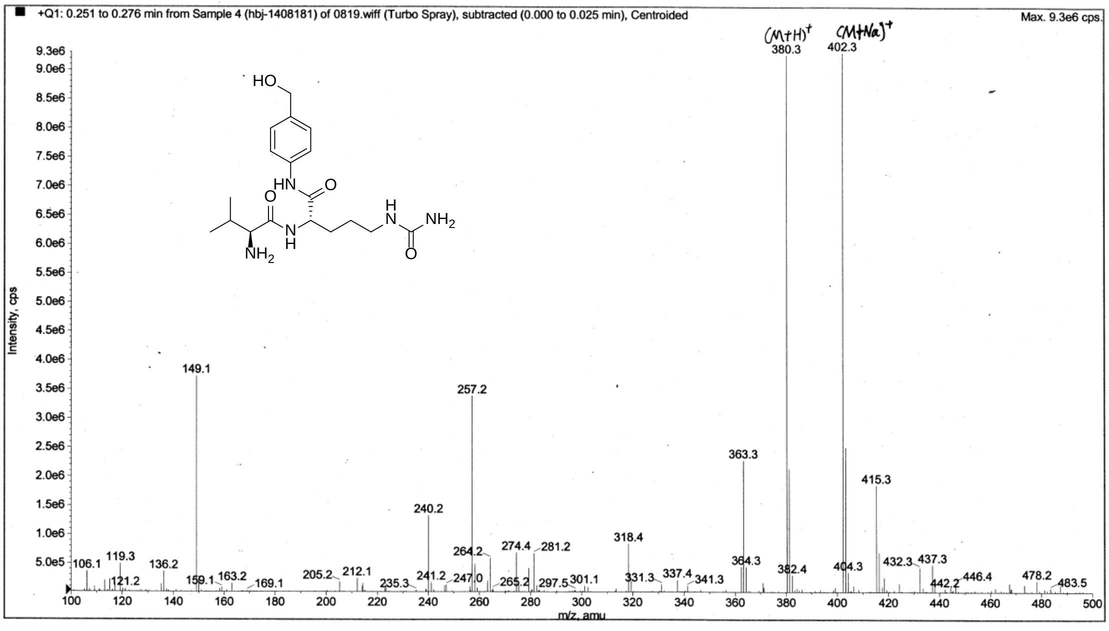

Figure S19. The ESI-MS spectrum of compound *9b*.

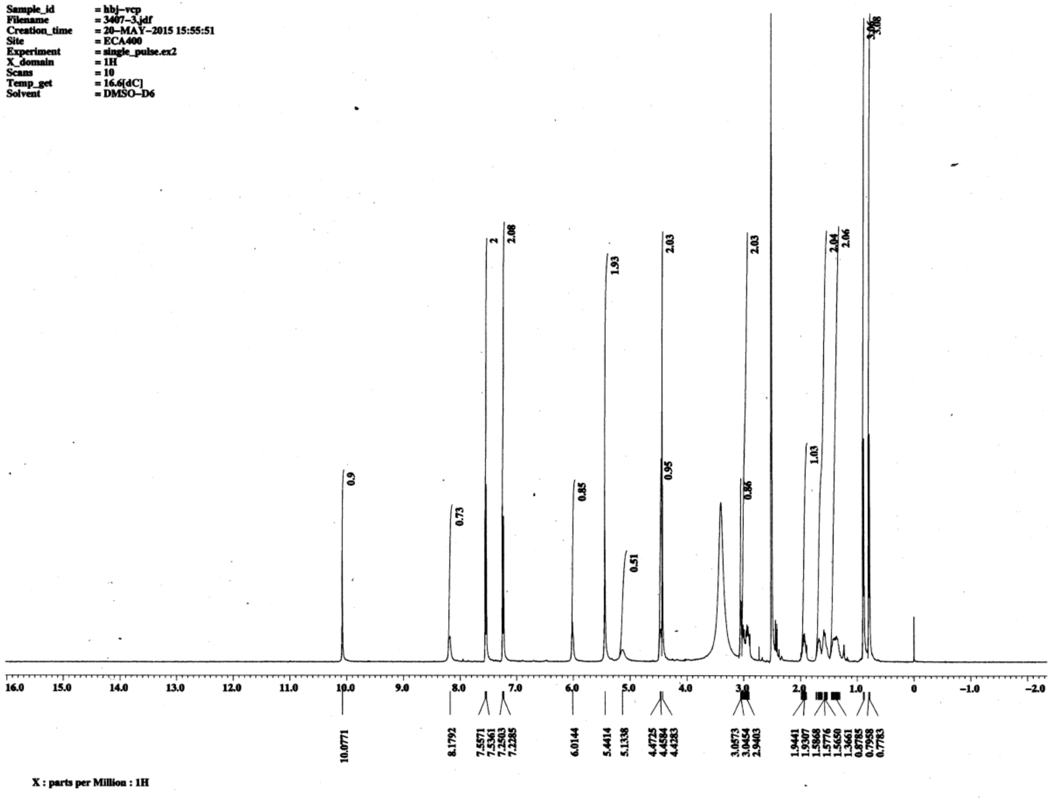

Figure S20. The ^{1}H-NMR spectrum of compound *9b*.

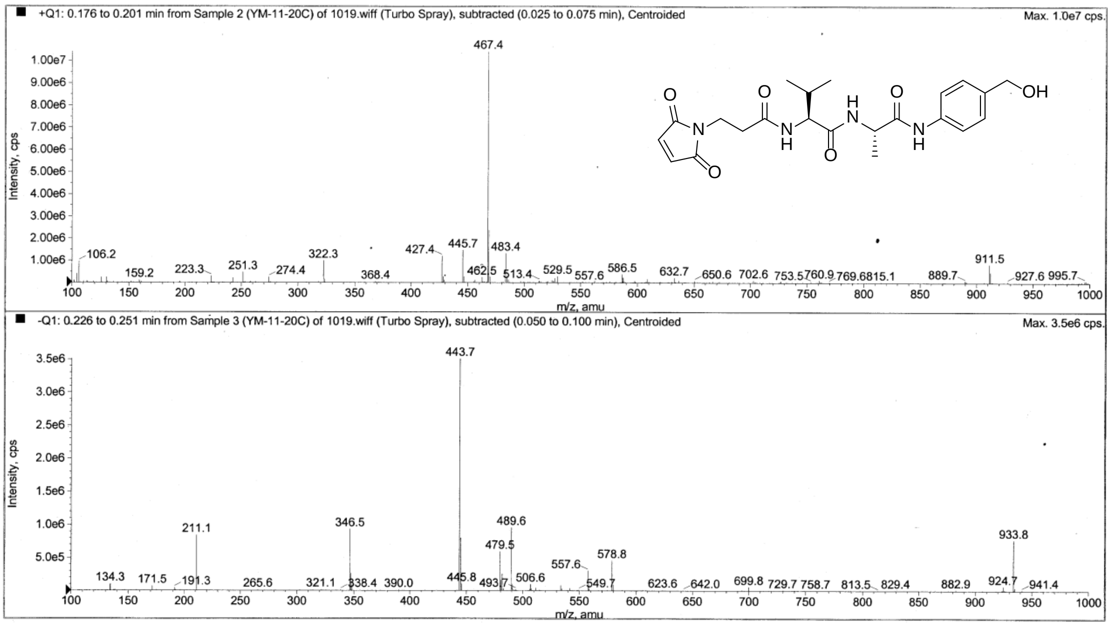

Figure S21. The ESI-MS spectrum of compound *10a*.

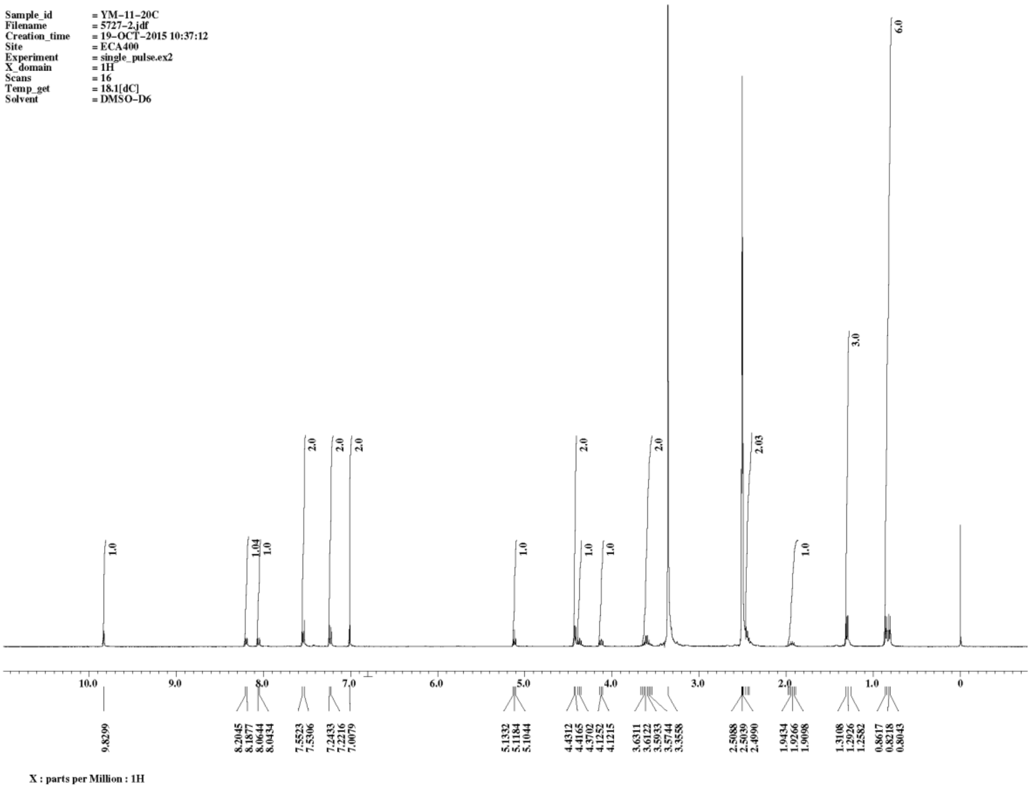

Figure S22. The ^{1}H-NMR spectrum of compound *10a*.

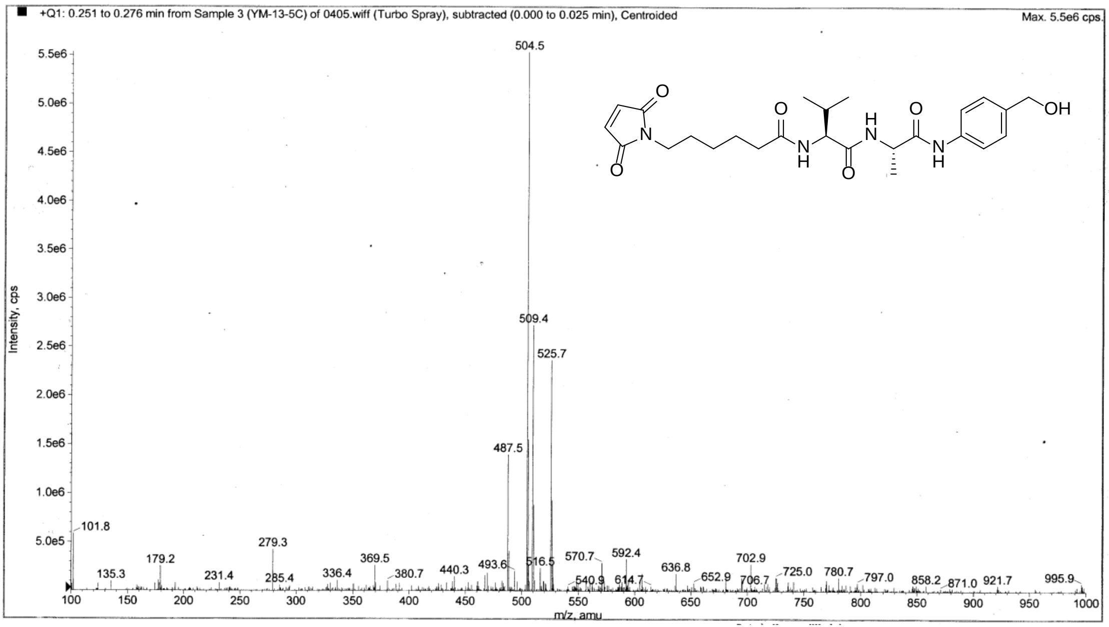

Figure S23. The ESI-MS spectrum of compound *10b*.

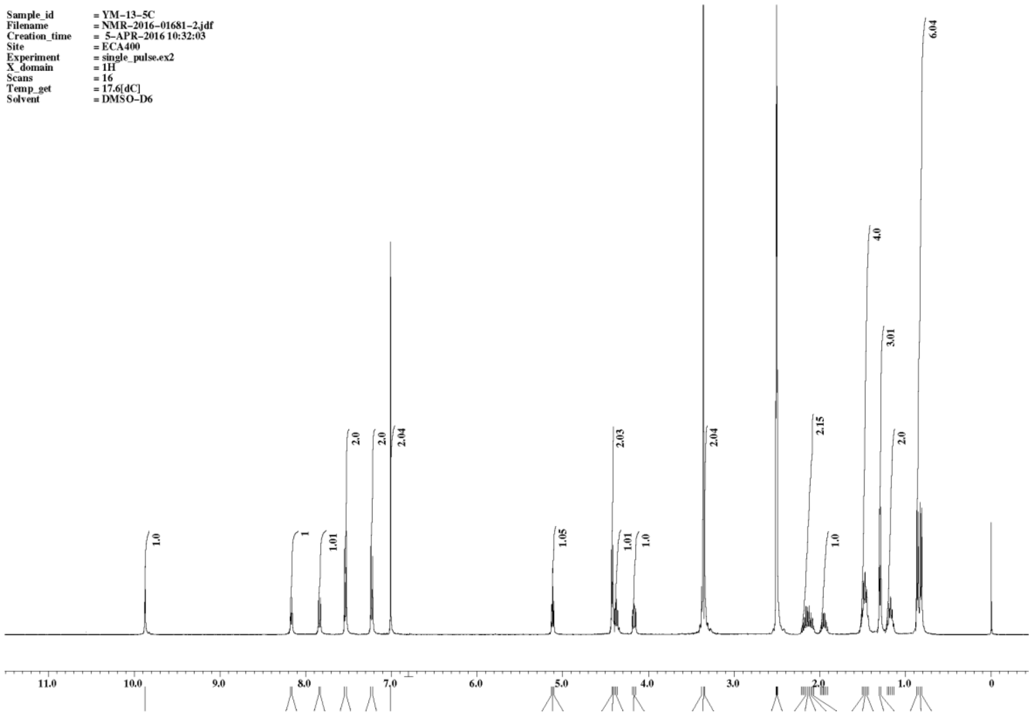

Figure S24. The ^{1}H-NMR spectrum of compound *10b*.

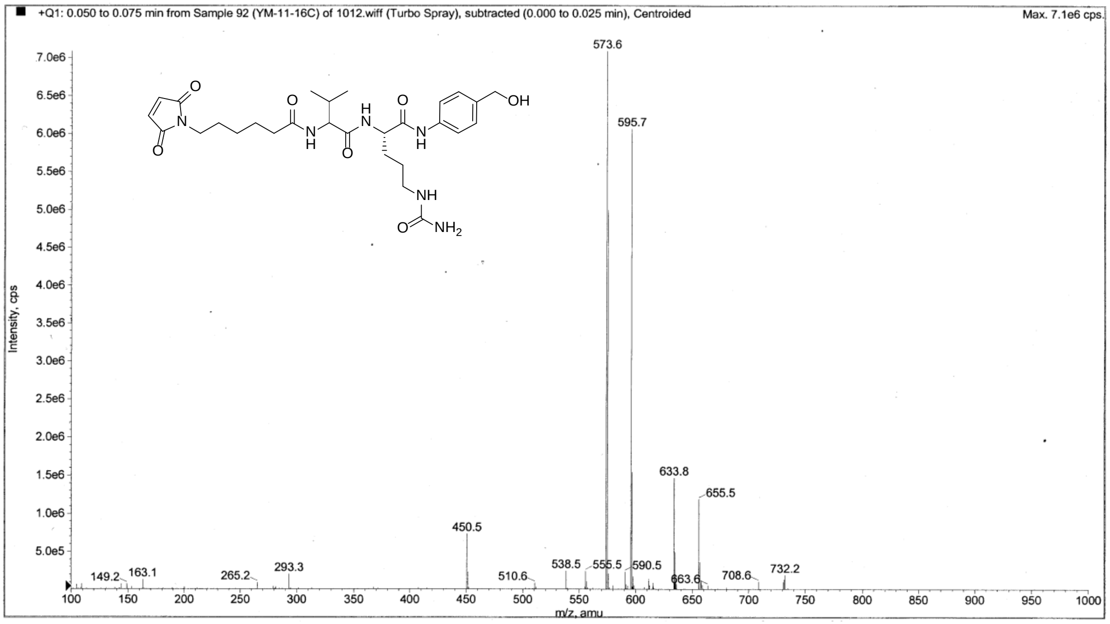

Figure S25. The ESI-MS spectrum of compound *10c*.

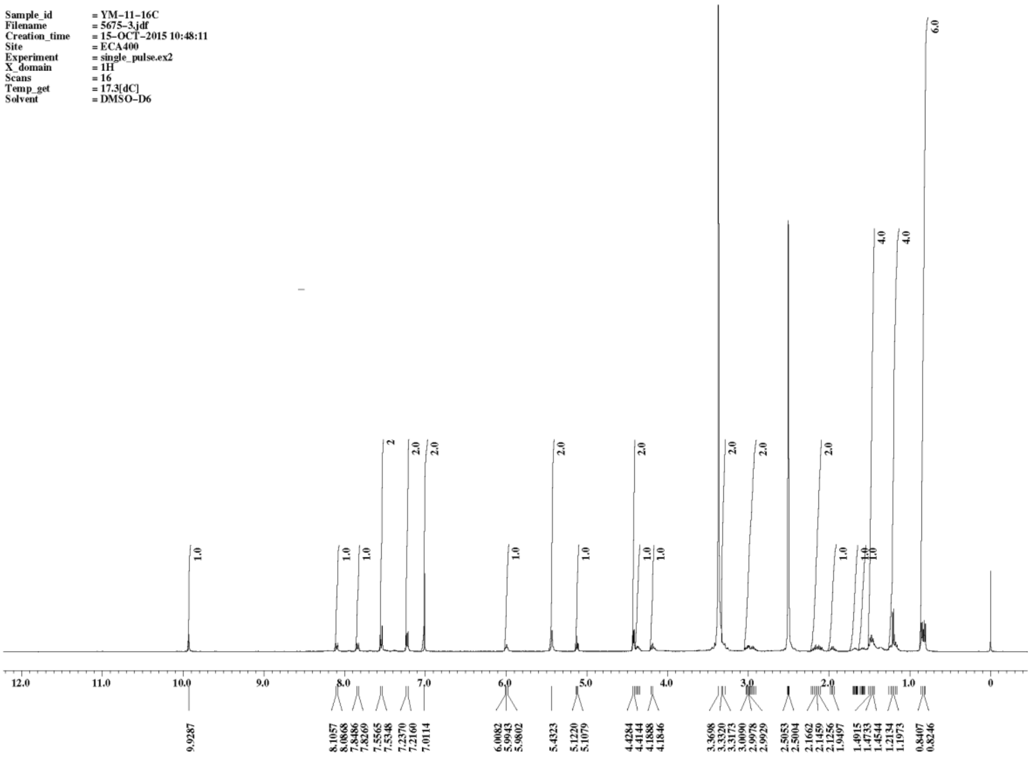

Figure S26. The ^{1}H-NMR spectrum of compound *10c*.

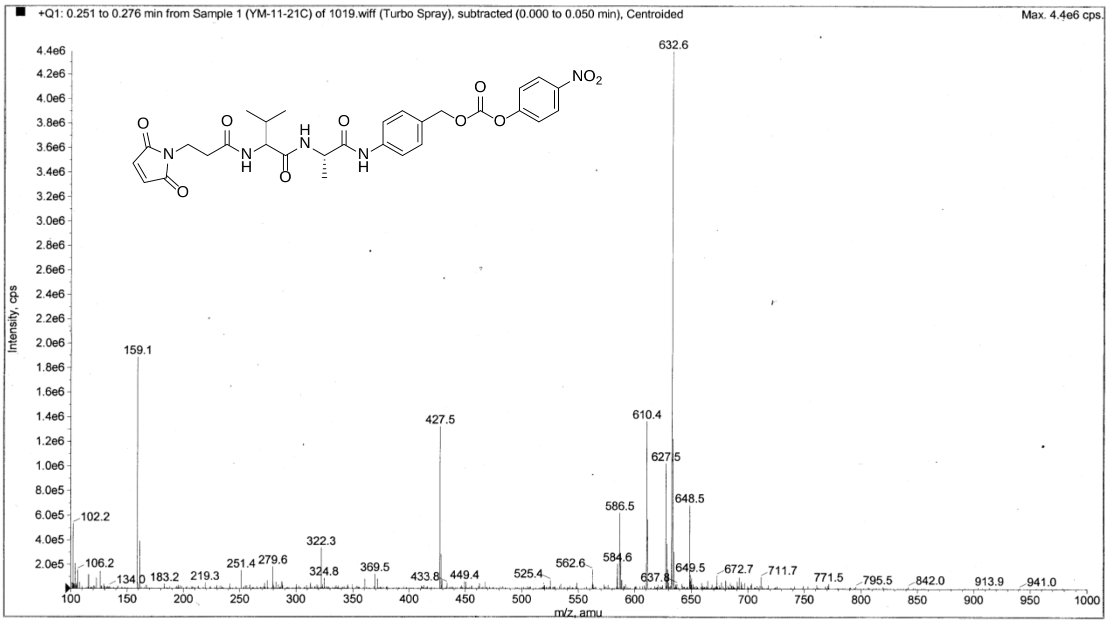

Figure S27. The ESI-MS spectrum of compound *11a*.

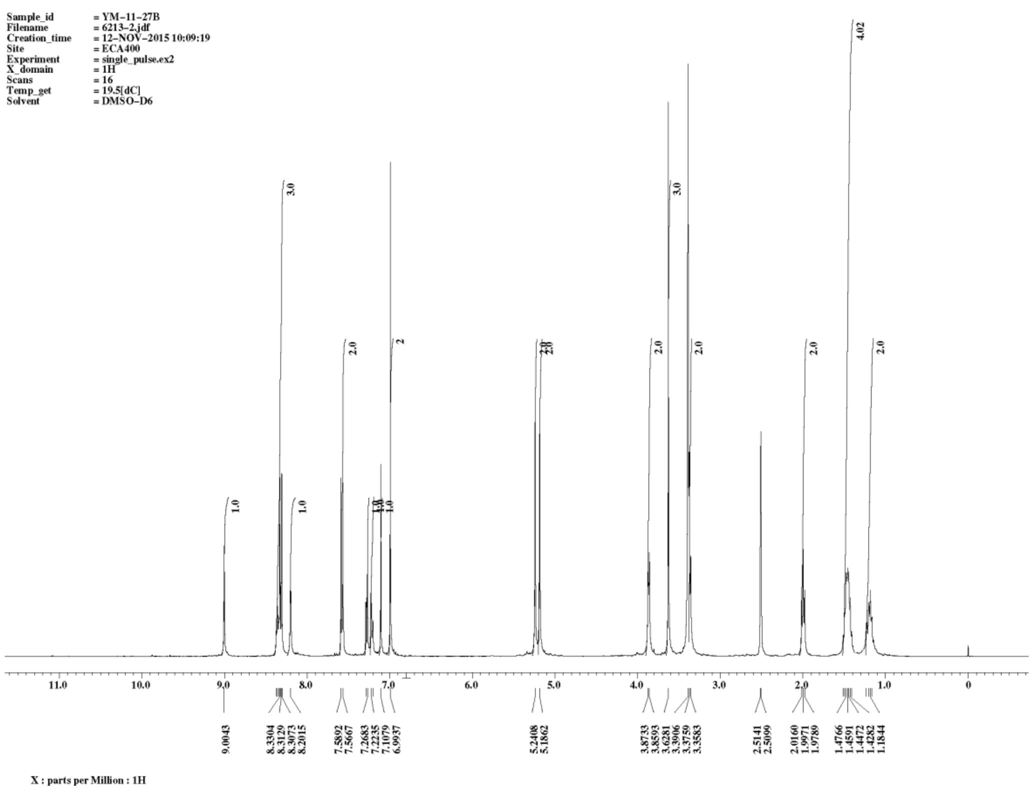

Figure S28. The ^{1}H-NMR spectrum of compound *11a*.

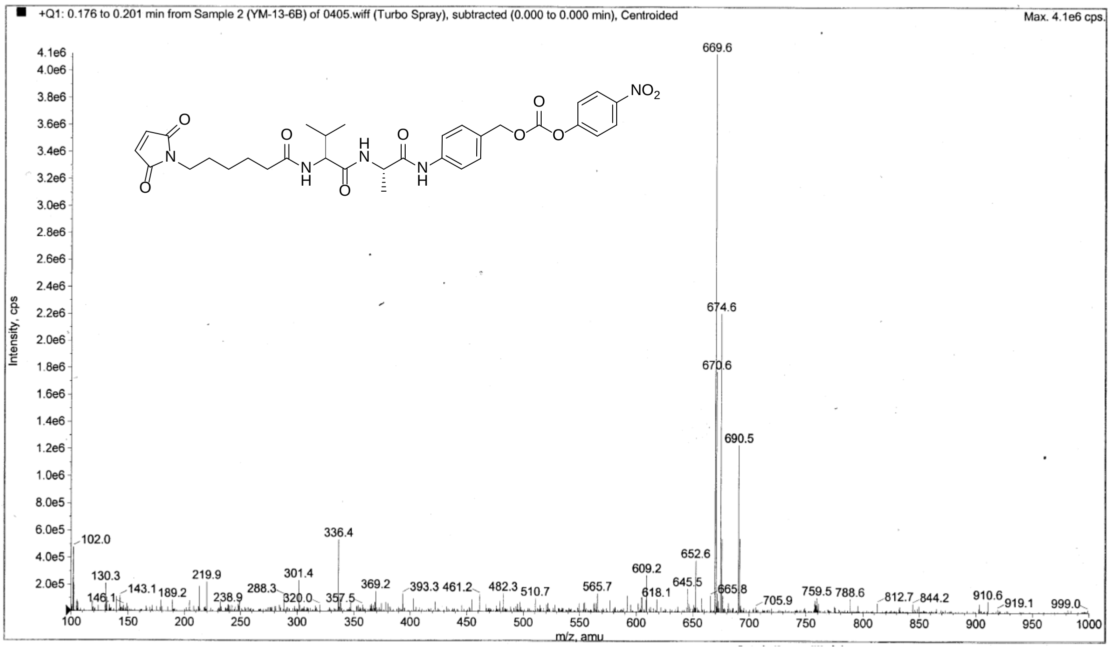

Figure S29. The ESI-MS spectrum of compound *11b*.

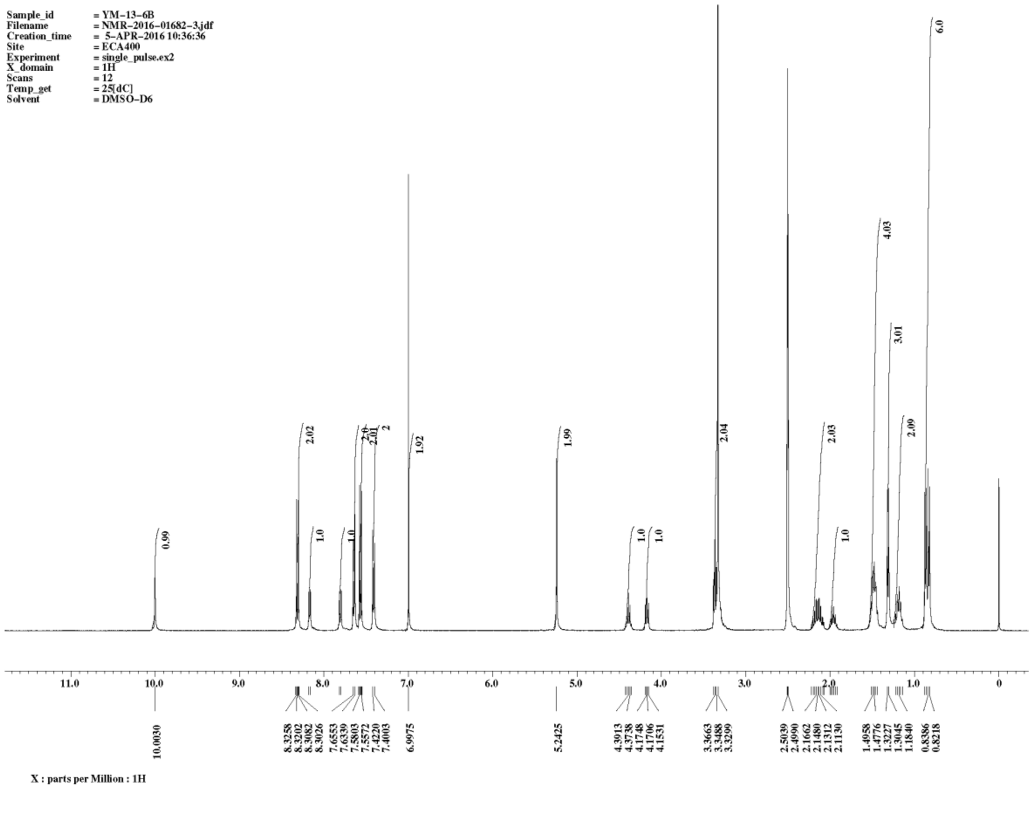

Figure S30. The ^{1}H-NMR spectrum of compound *11b*.

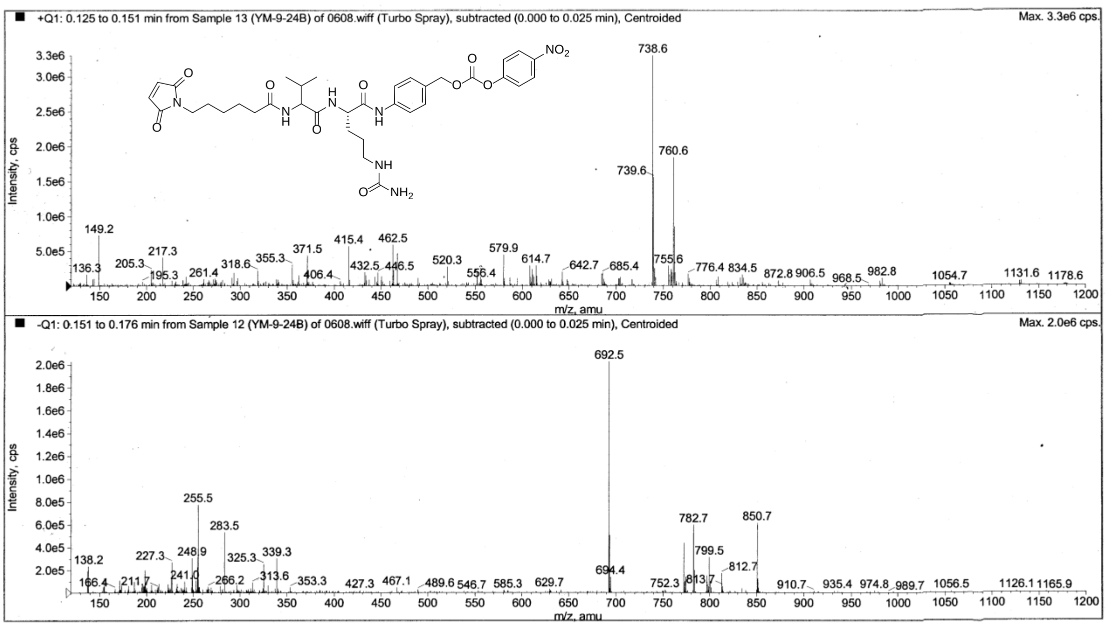

Figure S31. The ESI-MS spectrum of compound *11c*.

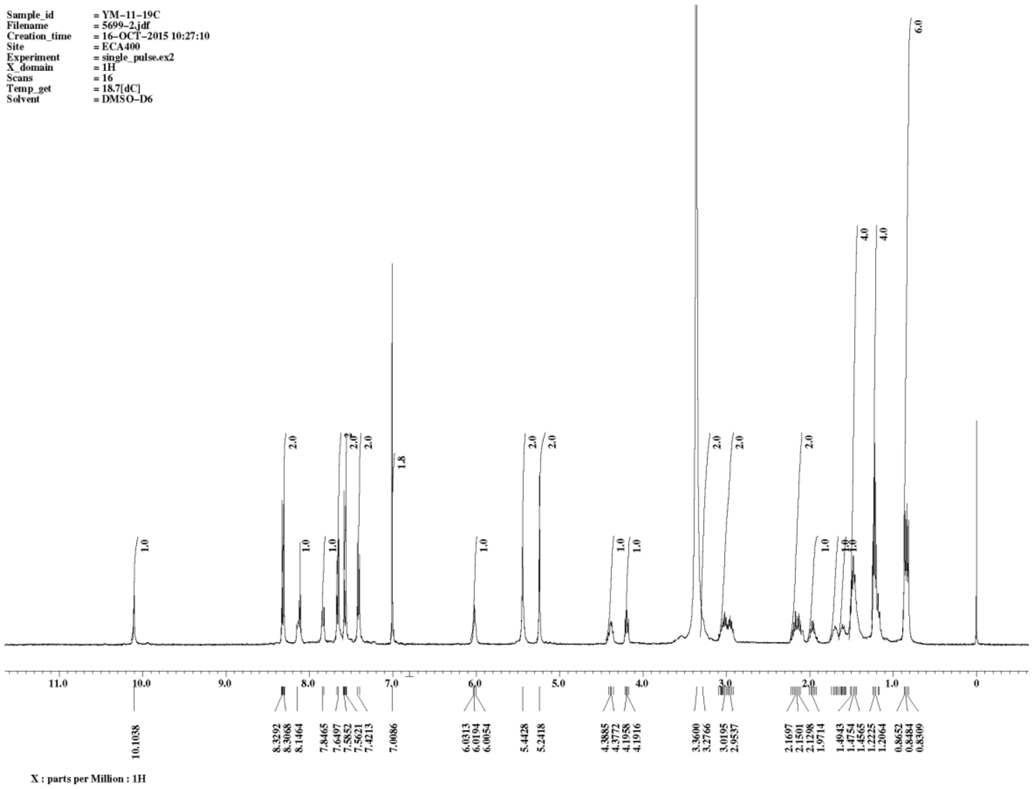

Figure S32. The ^{1}H-NMR spectrum of compound *11c*.

**Data Filename**	0639.d	**Sample Name**	YM-11-24
**Instrument Name**	TOF G6230A	**Acquired Time**	2017-02-10
**Acq Method**	YCL.M	**Acquired SW**	6200 series TOF/6500 series
**IRM Calibration Status**	**Success**		
**User Chormatograms**			

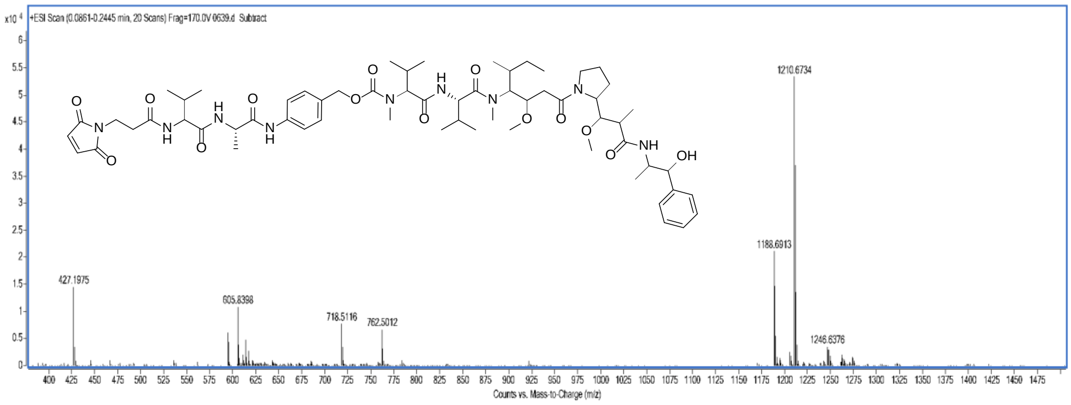

Figure S33. The ESI-MS spectrum of compound *12a*.

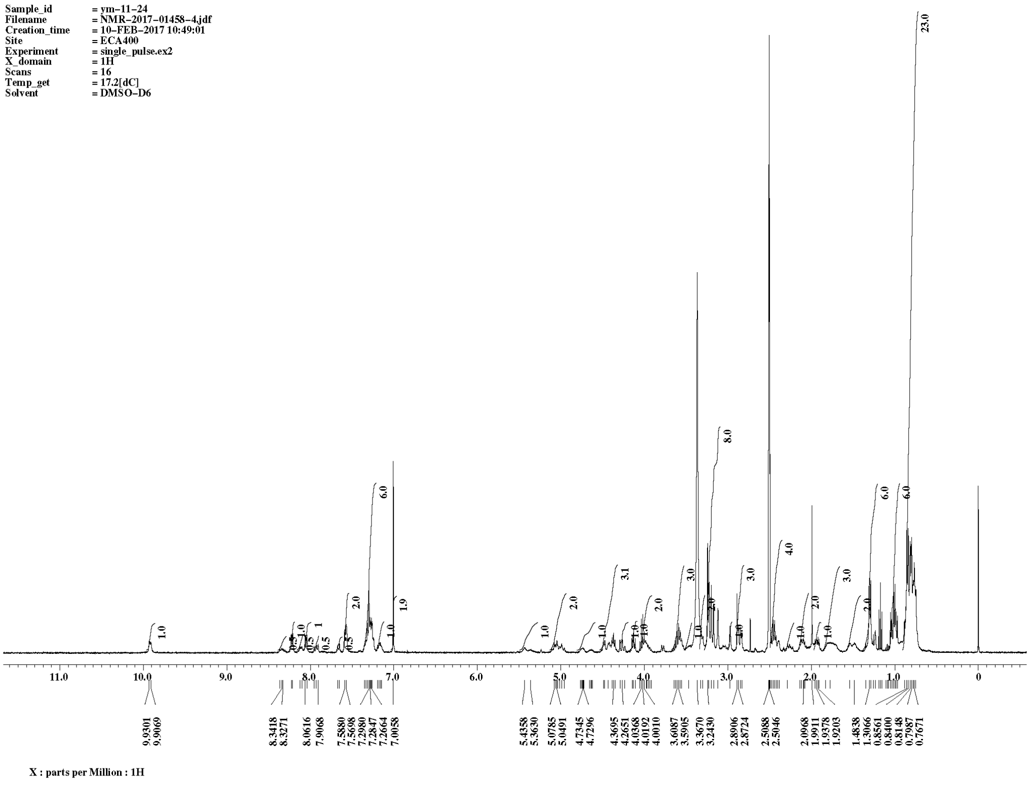

Figure S34. The ^{1}H-NMR spectrum of compound *12a*.

**Data Filename**	0640.d	**Sample Name**	YM-13-7
**Instrument Name**	TOF G6230A	**Acquired Time**	2017-02-10
**Acq Method**	YCL.M	**Acquired SW**	6200 series TOF/6500 series
**IRM Calibration Status**	**Success**		
**User Chormatograms**			

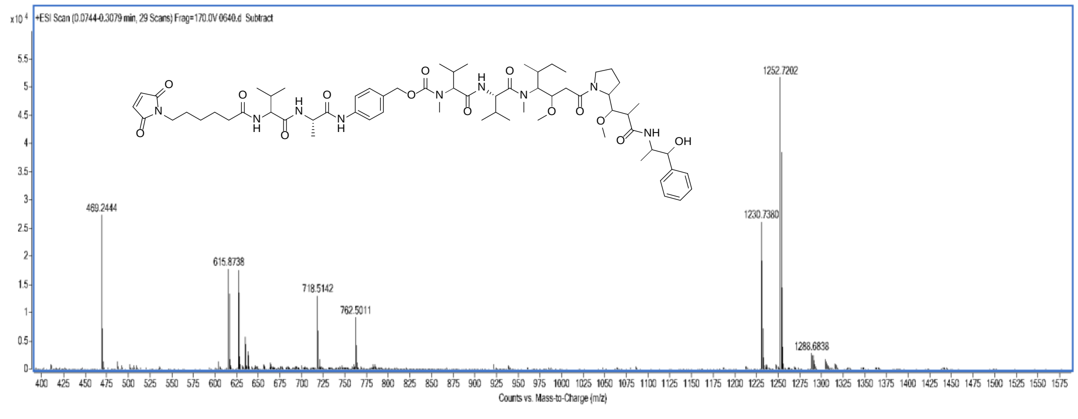

Figure S35. The ESI-MS spectrum of compound *12b*.

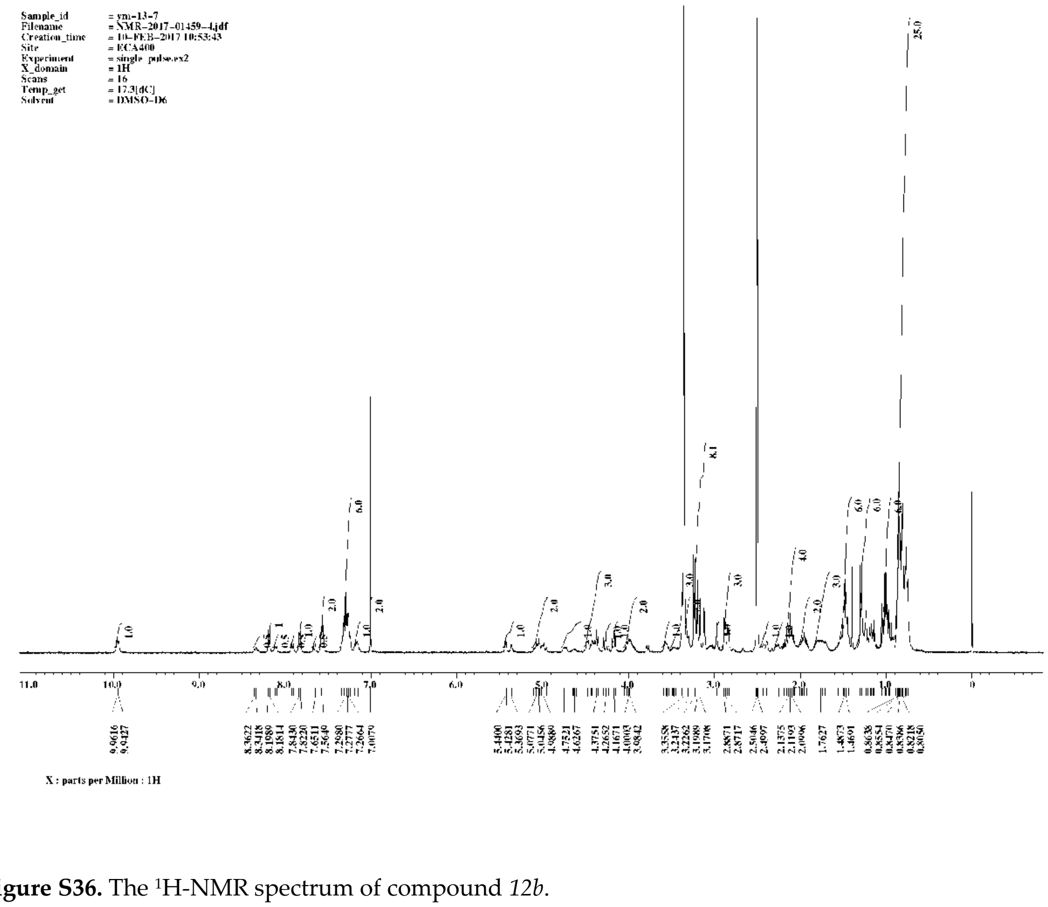

Figure S36. The ^{1}H-NMR spectrum of compound *12b*.

**Data Filename**	0642.d	**Sample Name**	YM-12-50
**Instrument Name**	TOF G6230A	**Acquired Time**	2017-02-10
**Acq Method**	YCL.M	**Acquired SW**	6200 series TOF/6500 series
**IRM Calibration Status**	**Success**		
**User Chormatograms**			

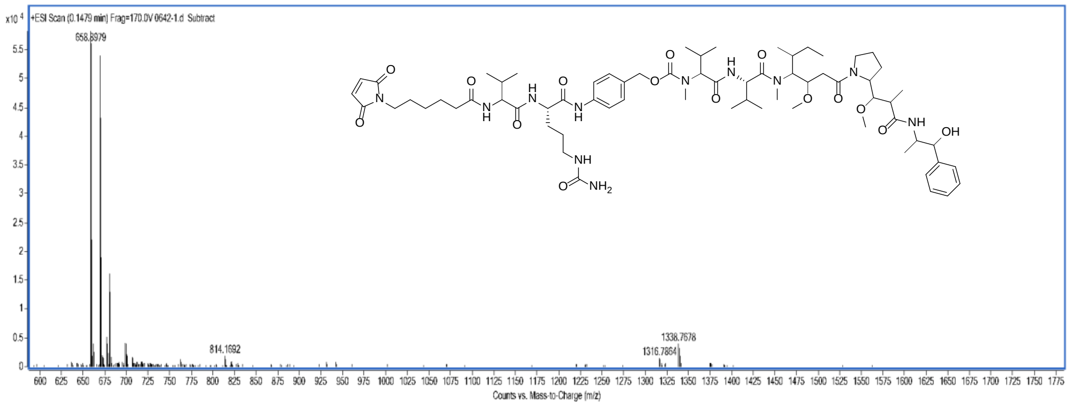

Figure S37. The ESI-MS spectrum of compound *12c*.

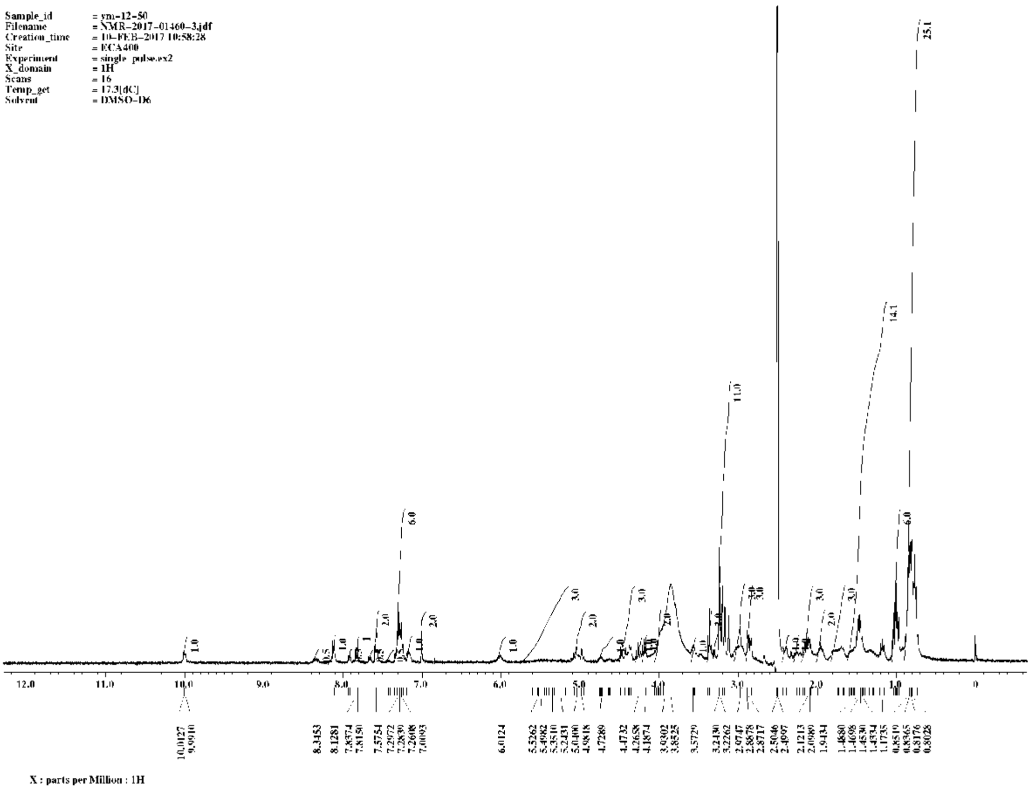

Figure S38. The ^{1}H-NMR spectrum of compound *12c*.
